# Zoogeography, taxonomy, and conservation of West Virginia’s Ohio River floodplain crayfishes (Decapoda, Cambaridae)

**DOI:** 10.3897/zookeys.74.808

**Published:** 2011-01-06

**Authors:** Zachary J. Loughman, Thomas P. Simon

**Affiliations:** 1 West Liberty University, Department of Natural Sciences and Mathematics, P.O. Box 295, West Liberty, West Virginia, 26074 USA; 2 SIndiana State University, Biology Department, 600 Chestnut Street, Science Building Room 281, Terre Haute, Indiana 47809 USA

**Keywords:** Crayfishes, West Virginia, Floodplain, *Cambarus*, *Orconectes*, *Fallicambarus*, *Procambarus*

## Abstract

The crayfish fauna of West Virginia consists of 23 species and several undescribed taxa. Most survey efforts documenting this fauna have been conducted in lotic waterways throughout the Appalachian plateau, Allegheny Mountains, and Ridge and Valley physiographic provinces. Bottomland forests, swamps, and marshes associated with large river floodplain such as the Ohio River floodplain historically have been under-surveyed in the state. These habitats harbor the richest primary burrowing crayfish fauna in West Virginia, and are worthy of survey efforts. In an effort to fill this void, the crayfish fauna of West Virginia’s Ohio River floodplain was surveyed from 2004 through 2009. From this survey, nine species from four genera were documented inhabiting the floodplain. Zoogeography, biology, and conservation status is provided for all nine crayfishes. The dominant genus along the floodplain is Cambarus, which includes Cambarus (Cambarus) carinirostris, Cambarus (Cambarus) bartonii cavatus, Cambarus (Procambarus) robustus and Cambarus (Tubericambarus) thomai. Cambarus (Tubericambarus) thomai is the most prevalent burrowing species occurring along the floodplain. The genus Orconectes consists of two native species, Orconectes (Cambarus) obscurus and Orconectes (Cambarus) sanbornii; and two invasive taxa, Orconectes (Gremicambarus) virilis and Orconectes (Procambarus) rusticus. Orconectes (Cambarus) obscurus has experienced a range extension to the south and occupies streams formerly occupied by Orconectes (Cambarus) sanbornii. Both invasive taxa were allied with anthropogenic habitats and disturbance gradients. The genera Fallicambarus and Procambarus are represented by a single species. Both Fallicambarus (Cambarus) fodiens and Procambarus (Orconectes) acutus are limited to the historic preglacial Marietta River Valley.

## Introduction

Crayfishes are among the most imperiled animal groups in North America (Taylor et al. 1996; Taylor et al. 2007; Taylor 1999; Taylor and Schuster 2004; Schuster 1997). Reasons for this imperilment vary from high levels of endemism and invasive species impacts to habitat destruction (Daniels et al. 2001; Hobbs et al. 1989; Lodge et al. 2000a, b). Invasive species in particular represent an important threat. Species such as Orconectes (Procericambarus) rusticus (Girard, 1852) and Orconectes (Gremicambarus) virilis (Hagen, 1870),can eliminate native species, resulting in lower species diversity and “biotic homogenization” of crayfish faunas (Lodge 1993). Documenting native crayfish communities prior to invasive species infestation or other impact from environmental stressors is important to crayfish conservation (Taylor et al. 2007).

West Virginia crayfishes have received moderate attention in the past century (Faxon 1914; Newcombe 1929; Jezerinac et al. 1995). The first report of West Virginia crayfishes was that of Faxon (1914), who listed two species in the state. Subsequent studies by Newcombe (1929) increased the number of species to 15; however, Jezerinac et al. (1995) provided the most thorough study of the state’s fauna, documenting 21 taxa. Recently West Virginia crayfishes have received a surge of research. Loughman (2007) increased the known crayfish fauna with the addition of Procambarus (Ortmannicus) acutus (Girard, 1852). Swecker et al. (2010) investigated the extirpation of Orconectes (Faxonius) limosus (Rafinesque, 1817) in the eastern panhandle of the state. Loughman et al (2009) provided natural history information for 11 of 24 species, while Loughman and Welsh (2010) reviewed the state’s fauna and reported Procambarus (Ortmannicus) zonangulus Hobbs & Hobbs, 1990, as another introduced species.

The focus of this study is on the floodplain and stream confluences with the Ohio River mainstem. This area is an ecological system that previous investigators neglected while surveying West Virginia’s crayfish. In addition, several potential conservation threats have occurred in the state since publication of Jezerinac et al. (1995). In response to these threats, a crayfish survey was initiated in the spring of 2004 along the Ohio River floodplain of West Virginia. The Ohio River’s importance as a trade route has attracted increased levels of industrialization and urbanization. The crayfish fauna inhabiting the Ohio River floodplain from Huntington, Cabell County; north to Chester, Hancock County, West Virginia, includes eight native species making it one of the most diverse crayfish faunas for a contiguous West Virginia habitat (Jezerinac et al. 1995). Another purpose of this survey was to document shifts in the Ohio River floodplain’s crayfish fauna since Jezerinac et al. (1995) and identify any biotic or abiotic threats to floodplain crayfish populations.

### Ohio River floodplain habitats

The North American large river floodplain is conducive to crayfish diversity due to the myriad of lentic and lotic habitats associated with these ecosystems (Trautman 1981; Hardin et al. 1989; Sparks 1995; Benke 2001). West Virginia’s portion of the Ohio River floodplain houses these systems and supports a diverse crayfish fauna. Lentic habitats present on the floodplain include swamps, marshes, ephemeral pools, and anthropogenically created habitats (e.g., roadside ditches). Burrowing crayfishes, especially primary burrowers use lotic habitats and their associated floodplains (Hobbs 1981; Taylor and Schuster 2004). Given the lack of fish present in ephemeral wetlands, these areas prove to be important nursery habitats for primary burrowers (Hobbs 1981). Tertiary burrowers also occur within the floodplain, occupying streams within these environments.

## Methods

### Study Site

**Figure 1.**
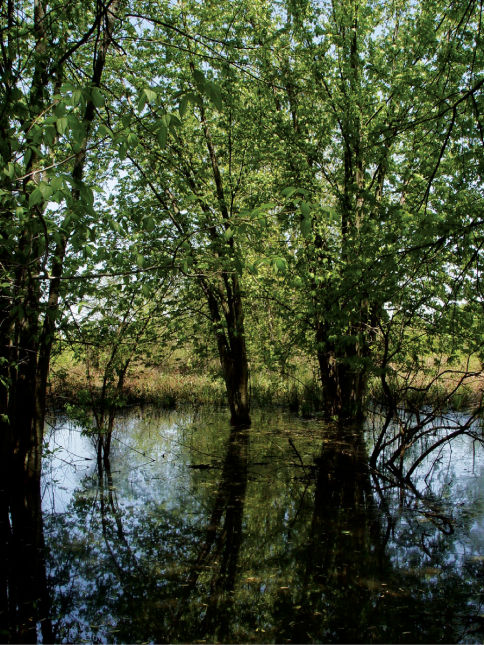
Ashton, Lower Ohio basin, Mason County, West Virginia – Red/Silver Maple Swamp. Maple swamps were the most prevalent bottomland habitats present on the Ohio River floodplain. Cambarus thomai were abundant in these situations.

Forests occurring along the Ohio River floodplain represent the most expansive bottomland forest in West Virginia. These habitats are characterized by nutrient rich, alluvial soils and vegetation adapted for seasonal inundation (Colburn 2004). Forest dominants include silver maples (Acer saccharinum, Marsh), red maples (Acer rubrum, L.) and black willows (Salix nigra, Marsh) (Strausbaugh and Core 1978). Nutrients are provided to soils by seasonal flooding events (Figure 1). These floods also inundate ephemeral wetlands present in low-lying sections of floodplain forest.

Active hydroperiod seasons typically last from January through early June (Hardin et al. 1989). During this period a multitude of invertebrates and vertebrates, including crayfish, utilize these wetlands for various aspects of their life history. A period of drawdown begins during the early summer months and by late June–July much of the floodplain’s ephemeral wetlands experience complete evaporation. Periodic summer storms occasionally reflood these wetlands, but the majority of pools remain dormant until the following fall or winter (Z. J. Loughman personal observation).

### Collection Methods

**Figure 2.**
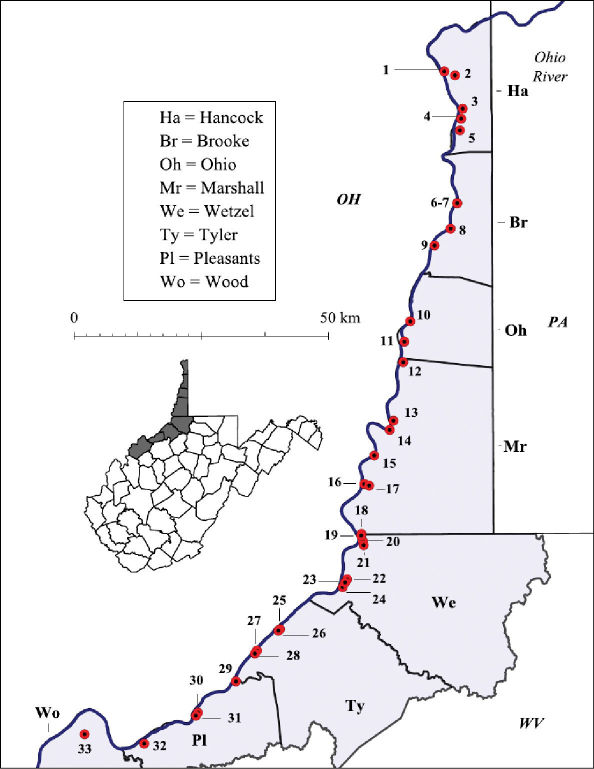
Ohio River floodplain collection sites – Northern Counties. Site numbers correspond to numbers in Table 1.

**Figure 3.**
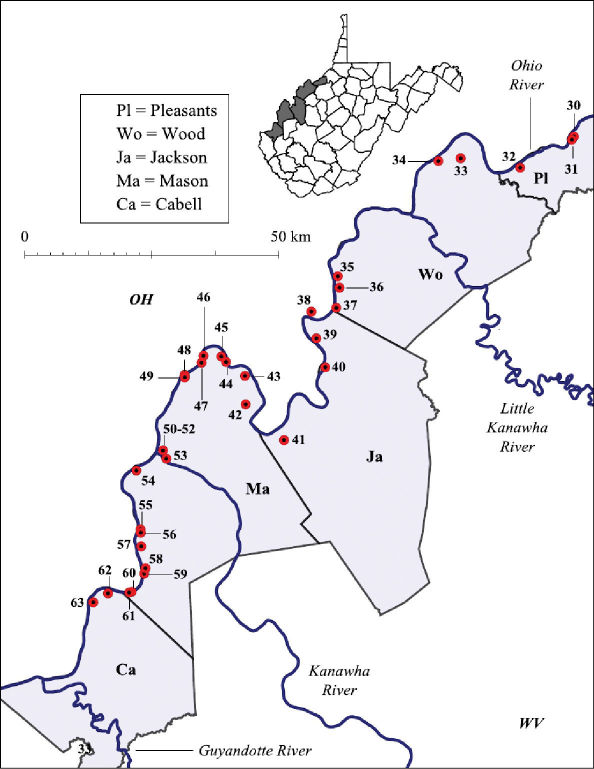
Ohio River floodplain collection sites – Southern Counties. Site numbers correspond to numbers in Table 1.

**Figure 4.**
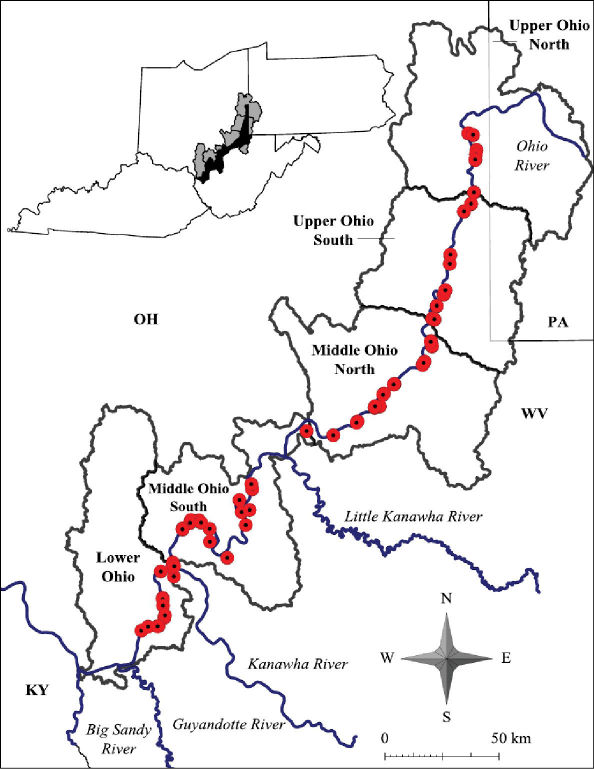
Ohio River floodplain collection sites – Major watersheds.

Both lentic and lotic habitats were surveyed to determine the crayfish diversity on the floodplain (Figures 2–4, and Table 1). All marshes, swamps, ephemeral wetland complexes and large roadside ditches from Huntington, Cabell County, to Chester, Hancock County, were assessed through trapping, dip netting, or burrow excavation. All large stream confluences were surveyed through trapping in deep water or seining. Headwater streams were evaluated through hand collecting and seining.

**Table 1.** West Virginia Ohio River Floodplain crayfish collection sites. Site numbers correspond to numbers in Figure 2 and 3.

Site #	Specific Location
1	Tomlinson Run backwater at RT 2 crossing, Hancock County, 40.54026 -80.628075
2	Hardin Run 0.81 km (0.5 mi) from CR 2-7/RT 2 intersection on CR 2-7, Hancock County, 40.533314 -80.60326
3	Kings Creek at RT 2 crossing, Hancock County, 40.435715 -80.592514
4	Nameless Tributary 0.03 km (0.02 mi) from CR 2-8/RT 2 intersection on CR 2-8, Hancock County, 40.4563 -80.589485
5	Holbert Run 1.61 km (1.0 mi) from CR 2-8/ Rt 2 intersection along CR 2-8, Hancock County, 40.474045 -80.58584
6	RT 2 crossing of nameless tributary in Beech Bottom, Brooke County, 40.306442 -80.5997
7	RT 2 crossing of nameless tributary 2.27 km (1.41 mi) S of Beech Bottom, Brooke County, 40.23163 -80.6523
8	Buffalo Creek at RT 2 crossing in Wellsburg, Brooke County, 40.261375 -80.61508
9	Cross Creek at entrance to Bruin Drive adjacent to Brooke High School, Brooke County, 40.306442 -80.5997
10	Short Creek at RT 2 crossing, Ohio County, 40.18312 -80.676865
11	Wheeling Creek at confluence of Ohio River in Wheeling, Ohio County, 40.063889 -80.72510
12	Boggs Run at RT 2 crossing, Marshall County, 40.02481 -80.72577
13	Little Grave Creek at RT 2 crossing in Moundsville, Marshall County, 39.920944 -80.748566
14	Big Grave Creek at Ohio River confluence in Moundsville, Marshall County, 39.9046 -80.75731
15	Nameless tributary at RT 2 crossing adjacent to Columbia Chemical , Marshall County, 39.85933 -80.79305
16	Fish Creek at RT 2 crossing, Marshall County, 39.808643 -80.81616
17	Long Run at Long Run/Fish Creek confluence, Marshall County, 39.805878 -80.8052
18	PPG Wildlife Management Area adjacent to RT 2 S, Marshall County, 39.736244 -80.84638
19	Ohio River backwater at Marshall/Wetzel County line, 39.717846 -80.514959
20	Proctor Creek at RT 2 crossing, Wetzel County, 39.70037 -80.81791
21	RT 2 crossing of nameless tributary at Marshall/Wetzel County line, 39.720192 -80.82281
22	Doolins Run at RT 2 crossing, Wetzel County, 39.639576 -80.85607
23	Fishing Creek at RT 2 crossing, Wetzel County, 39.63576 -80.85848
24	Maple Swamp adjacent to RT 2 S in New Martinsville, Wetzel County, 39.32582 -80.866234
25	Cow House Run at RT 2 crossing, Tyler County, 39.551327 -81.01001
26	Narrows Run at RT 2 crossing 1.75 km (1.09 mi) S of Sistersville, Tyler County, 39.54874 -81.013626
27	Maple swamp adjacent to RT 2 S in Friendly, Tyler County, 39.50822 -81.06736
28	Nameless tributary at RT 2 crossing in Friendly, Tyler County, 39.513386 -81.06285
29	Ben’s Run at RT 2 crossing, Pleasants County, 39.46337 -81.08457
30	Ohio River embayment 4.03 air km (2.52 mi) S of St. Mary’s, Pleasants County, 39.397575 -81.202415
31	Middle Island Creek at RT 2 crossing, Pleasants County, 39.40328 -81.197624
32	Vernal pool adjacent to RT 2 N across from Cytec Community Fishing Area, Pleasants County, 39.347824 -81.32024
33	Big Run at CR 21-1 crossing, Wood County, 39.364048 -81.45656
34	Boaz Swamp Wildlife Management Area, Wood County, 39.462868 -81.10855
35	Lee Creek at CR 11 crossing, Wood County, 39.153275 -81.73507
36	Bellville Wildlife Management Area 4.03 km (2.50 mi) S of Bellville, Wood County, 39.132915 -81.730865
37	Nameless tributary crossing 3.54 km (2.2 mi) S of Parkersburg, Wood County, 39.05142 -81.742836
38	Vernal pool adjacent to railroad tracks 3.56 km (2.21 mi) N of Ravenswood, Jackson County, 39.09015 -81.79469
39	Flooded field adjacent to RT 33 S 9.72 air km (6.04 mi) N of Ravenswood, Jackson County, 39.04274 -81.7827
40	Little Sandy Creek at intersection of RT 68/CR 8, Jackson County, 38.991497 -81.761765
41	Little Mill Creek at crossing of RT 33 N 9.43 air km (5.86 mi) N of Ravenswood, Jackson County, 38.86171 -81.85407
42	West Creek at intersection of CR 12 /CR 10, Jackson County, 38.924362 -81.94200
43	Roadside ditch adjacent to RT 62 S at Mountaineer Power Plant, Mason County, 38.974934 -81.94418
44	Sliding Creek at intersection of CR 4/RT 33, Mason County, 38.999382 -81.987686
45	Red-Osier Dogwood swamp adjacent to RT 33 S in Hartford, Mason County, 39.008915 -81.99847
46	Slough adjacent to RT 33 N in Mason, Mason County, 39.00983 -82.03899
47	Roadside ditch adjacent to RT 62 N 0.54 km (0.34 mi) S of Clifton, Mason County, 38.997456 -82.04335
48	Roadside ditch adjacent to RT 62 N 0.34 km (0.21 mi) N of Hallwood, Mason County, 38.97562 -82.081314
49	Roadside ditch adjacent to RT 62 N 1.47 km (0.91 mi) N of Lakin, Mason County, 38.971046 -82.08092
50	Large vernal pool 0.24 km (0.15 mi) E of Krodell Park, Mason County, 38.84098 -82.12836
51	Vernal pool complex at RT 2/Lighthouse Gospel Church Road intersection, Mason County, 38.82201 -82.13136
52	Krodel Park marsh adjacent to Fort Randolph reproduction, Mason County, 38.785404 -82.12209
53	Pin oak swamp adjacent to Point Pleasant Moose Lodge in Wagner, Mason County, 38.833603 -82.12227
54	Roadside ditch adjacent to RT 2 9.17 km (5.7 mi) S of Point Pleasant, Mason County, 38.80469 -82.18821
55	Roadside ditch adjacent to RT 2 N 0.90 air km (0.56 mi.) N of Hogsett, Mason County, 38.70056 -82.17708
56	Roadside ditch adjacent to RT 2 N 0.22 air km (0.14 mi) N of Hogsett, Mason County, 38.694496 -82.1765
57	Pasture field 3.54 km (2.2 mi) N of Robert C. Byrd Dam entrance, Mason County, 38.67026 -82.174995
58	Roadside ditch adjacent to RT 2 N 1.93 km (1.2 mi) N of Glenwood, Mason County, 38.58816 -82.201004
59	Maple swamp adjacent to RT 2 railroad crossing in Ashton, Mason County, 38.622005 -82.16758
60	Roadside ditch adjacent to RT 2 N 2.91 km (1.81 mi) N of Clover, Mason County, 38.589428 -82.19548
61	Ditch adjacent to RT 2 N 1.96 air km (1.22 mi) N of Greenbottom, Cabell County, 38.570004 -82.28176
62	Green Bottom Swamp at Hoeft Marsh Wildlife Management Area, Cabell County, 38.58616 -82.24878
63	Roadside ditch adjacent to RT 2 N in Greenbottom, Cabell County, 38.570001 -82.28176

### Burrowing crayfish collecting methods – trapping in surface waters

Collapsible minnow traps were the chief collecting method used for this study. Collapsible minnow traps were preferred over classic metal minnow traps because of their ease of storage and manipulation in the field, larger entrance portals, and rate of degradation by natural predators (e.g., turtles, mammals) in the event of trap loss (Z. J. Loughman, personal observation). Entrance portal diameter has been shown to bias capture rates for various crayfish, sizes warranting the use of collapsible traps with larger portals (Huner and Espinoza 2004). Traps were placed in all roadside ditches, swamps, marshes, ponds, Ohio River embayments, and ephemeral wetlands along the floodplain (n = 31 sites).

Traps were placed in water bodies during mid- to late-January during 2004 and 2005, and checked biweekly January through April. During this sampling period, surface activity of primary and secondary burrowing crayfishes increase; making crayfish community analysis more efficient than in other seasons for these behavioral groups (Hobbs 1981; Taylor and Anton 1998; Simon 2001). This method proved to be efficient as large numbers of burrowing crayfishes were obtained in a very short amount of time.

### Burrowing crayfish collecting methods – excavation

Burrowers were also collected by excavation. Burrow activity was determined by the presence of chimneys or fresh mud pellets at burrow portals. Active burrows were excavated with trowels and shovels until enlarged ”resting chambers” were reached (Hobbs 1942; Hobbs 1981). Once the resting chamber was breached, burrows were filled with water and plunged with the investigator’s hand and arm. This pumping action was usually enough to dislodge crayfish hiding within the confines of the burrow, drawing them into the resting chamber where they were grasped.

If initial plunging efforts were not successful in dislodging crayfish, the burrow was left undisturbed for several minutes. Crayfish, curious of this disturbance, often rose to the water/air interface where the waving of their antennae was observed. In this situation crayfish were quickly pinned to the sides of the burrow and extracted (Hobbs 1942). Burrow morphology data were collected on burrows containing crayfish that were not destroyed during the excavation process; data collected included central shaft depth (earths surface to dorsal surface of resting chamber), resting chamber width and height, terminal burrow depth (earths surface to ventral surface of deepest chamber), and burrow contents. All measurements were in centimeters.

### Burrowing crayfish collecting methods – nocturnal searches

Nocturnal searches were also employed, specifically to collect Procambarus acutus. This species is a secondary burrower that is active in ephemeral surface waters prior to drawdown (Page 1985). Random searches were initiated at least two hours after sunset to ensure nocturnal activity had commenced. Headlamps were used to illuminate crayfish foraging in thalwegs and littoral regions of ephemeral pools. Crayfish were often initially observed by their eyes reflecting light (Hobbs 1942; Hobbs 1981). Once observed, crayfish were collected by hand or with dipnets.

Burrowing species were also collected from burrows at night. Crayfish were observed at their burrow entrances with their chelae and antennae resting at the burrow/atmosphere interface. In the capture attempt, crayfish were quickly pinned to the sides of their burrows. They were easily approached if indirect light was used but when direct light made contact with them they quickly retreated to the deepest regions of their burrows. Care was taken not to grasp the crayfish by its chelae, which were readily autotomized.

### Stream crayfish collecting methods

The primary collection method used for stream species were seines. Seines were setup at the terminal ends of riffles, runs, and glides in first through sixth order streams. By disturbing the stream’s substrate, crayfish were dislodged from their cover and flowed downstream into the positioned seine. At each stream site (n = 31) a minimum of five seine haul efforts (a single seine haul = one seining effort) and maximum of 10 seine hauls were performed. Effort was increased with increasing stream size and habitat complexity.

Leaf packs were surveyed in stream pools using long-handled, sturdy bait well dip nets. These were used to “shovel” leaf packs onto a minnow seine that was spread out on the stream bank. Crayfish were then picked from the collected leaf pack on shore. After they were removed from the leaf pack it was returned upstream of its original location so the contents could again be used by the stream’s benthos. All crayfish life stages utilized leaf packs, making this method extremely important for determining reproductive success and recruitment.

### Data collection

Data sheets and field jar labels were completed for each site surveyed (Simon 2004). Vouchered crayfish were preserved in 70% ethanol and identified in the laboratory using Hobbs (1989) and Jezerinac et al. (1995). Morphometrics were taken with digital calipers on all preserved crayfish following Hobbs (1942, 1981). Measurements (mm) included carapace length (TCL), palm length (PL), areola width (AW), and areola length (AL). Crayfish were sexed, and the reproductive condition of each individual determined, following Hobbs (1981). Ovigerous females and females with pleopodal instars were transported back to the laboratory, where the total number of instars for each female was determined. Maladies (regenerated chelae, missing chelae, chelae scars, etc.) were noted for each crayfish. Museum numbers refer to specimen collections maintained at the West Liberty University (WLU) Astacology Collection.

### Conservation Ranks

All conservation ranks were determined following Nature Serve’s conservation ranking criteria (Master et al. 2009).

### Explanation of Species Accounts

The following section provides accounts for each species encountered along the West Virginia Ohio River floodplain. Descriptions, morphometrics, natural history and habitat, distribution, and conservation are discussed for each taxon. A description of the information content emphasized for each subheading is explained below.

### Diagnosis and Color in life

The diagnosis section describes morphological characters for each of the species. Characters and information content that uniquely identify each species are included. Specific color patterns and geographic morphs unique to the Ohio River floodplain are provided.

### Morphometrics

Morphometric data specific to animals captured during the survey are discussed. Total carapace lengths (TCL) for the largest male and female of each species are indicated. Morphometric tables are presented for each species and contain mean, range, and standard deviation for carapace length, palm length, areola width, and areola length for all specimens.

### Distribution

Distribution of each taxon encountered in the floodplain study area is discussed relative to previous survey efforts. Most of this discussion is a comparison of results observed by Jezerinac et al. (1995) with sites surveyed during this study. Distribution maps for each species are provided and represent sites surveyed during this effort only.

### Natural History and Habitat

Ecological observations for each species, including burrowing ability and habitat preferences, are described for each taxon. For primary and secondary burrowers, specific burrow usage and burrow morphology and architecture, as well as surface water usage, are described. Lentic and lotic habitats used by stream species are noted and specific microhabitats utilized are identified. Seasonal shifts in habitat usage and ontogenetic niche shifts are also described in this section. All observed species-specific behaviors are identified and discussed.

### Conservation Status

Current conservation standing and potential mechanisms of imperilment are identified and discussed following Master et al. (2009). Recommendations are made for taxa in need of conservation efforts and future monitoring.

## Results, dichotomous key, and species accounts

### Key to Form I Male Crayfishes of the West Virginia Ohio River Floodplain

**Figure 5.**
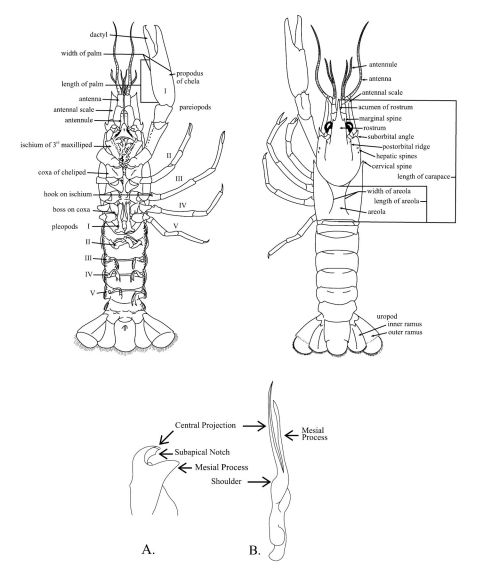
Schematic diagram of generalized male crayfish illustrating structures and measurements referred to within the key. **A** Cambarus gonopod **B** Orconectes gonopod. Taken from Hobbs 1989.

(See Figure 5 for terms and measurements)

**Table:** 

1.(a.)	Gonopod terminating in more than 2 elements; areola obliterated; chelae elongate; branchiostegal region of cephalothorax tuberculate	Procambarus (Orconectes) acutus
1.(b.)	Gonopod terminating in 2 terminal elements	2
2.(a.)	Gonopod containing two straight terminal elements; marginal spines always present	3
2.(b.)	Gonopod terminal elements bent 90° to central shaft; rostrum without marginal spines	6
3.(a.)	Gonopod terminal element length 40% or less than total central projection length; terminal elements short	4
3.(b.)	Gonopod terminal element length 50% or more than total central projection length; terminal elements long	5
4.(a.)	Shoulder present on cephalic base of central projection	Orconectes (Cambarus) obscurus
4.(b.)	Shoulder absent on cephalic base of central projection	Orconectes (Cambarus) sanbornii
5.(a.)	Mandible margin dentate; gonopod bent 30°; chelae green with two rows of yellow tubercles on mesial margin of palm; carapace lacking rusty spots	Orconectes (Gremicambarus) virilis
5.(b.)	Mandible margin entire; gonopod straight; chelae tips encircled with black band; rust colored spot on posterior branchiostegial region of cephalothorax	Orconectes (Procambarus) rusticus
6.(a.)	Opposable surface of dactyl (movable finger) of chela deeply notched; dactyl/chelae junction setiferous; brown wedge present on abdomen	Fallicambarus (Cambarus) fodiens
6.(b.)	Opposable surface of dactyl lacking notch; chelae non-setiferous; brown wedge absent	7
7.(a.)	Mesial margin of palm with disorganized tubercles; rostrum ventrally deflected; areola obliterated; coloration brown, amber, or bluish	Cambarus (Tubericambarus) thomai
7.(b.)	One or two rows of tubercles on mesial margin of palm; areola open	8
8.(a.)	Palmar tubercles in single adpressed row; fourth tubercle on opposable surface of fixed finger of propodus enlarged	Cambarus (Cambarus) carinirostris
8.(b.)	Palmar tubercles in double row, well defined; fourth tubercle on opposable surface of fixed finger of propodus not enlarged	9
9.(a.)	Palmar tubercles increasing in size from anterior to posterior portions of palm; rostrum margins thickened; rostrum blunt; overall color tan-brownish	Cambarus (Cambarus) bartonii cavatus
9.(b.)	Palmar tubercles uniform in size; rostral margins not thickened; rostrum acuminate; cephalothorax color pink-green; chelae green	Cambarus (Procambarus) robustus

### Cambarus (Cambarus) carinirostris

Hay, 1914

Cambarus bartonii carinirostris Hay 1914: 384. Taylor et al. 1996:29.
Cambarus (Cambarus) bartoni carinirostris Ortmann 1931:107.
Cambarus (Cambarus) bartonii carinirostris Hobbs 1969: 109, fig. 19m; 1974b:11, fig. 24; 1989:13, fig. 30. Thoma 1982:875. Thoma and Jezerinac 1982: 136. Jezerinac et al. 1995:76–83, fig. 35A–35H.
Cambarus (Cambarus) carinirostris Thoma and Jezerinac 1999: 97, fig 2, A–C. Loughman et al. 2009:226. Loughman 2010:39, fig. 36.
Cambarus carinirostris Taylor et al. 2007:382.

#### Diagnosis.

Rostrum broad, margins thickened and parallel, terminating in a 90° angle cephalically to form acumen; acumen consisting of a single upturned spiniform tubercle; median carina absent in floodplain populations; postorbital ridges truncate, cephalic margin with a weak tubercle; cephalothorax slightly flattened dorsoventrally in profile; 2–5 punctations across narrowest region of areola; branchiostegal region moderately punctate, with small tubercles; chelae broad and robust; mesial surface of palm with a single row of 5–7 adpressed tubercles; two prominent subpalmar tubercles present; enlarged 3_rd_ tubercle on mesial surface of fixed finger of propodus; first form gonopods contiguous at base, with 2 terminal elements bent 90° to base; central projection with subapical notch; total length of central projection equal to length of mesial process; mesial process bulbous, truncating distally; second form gonopod non-corneous and blunt; annulus ventralis rhomboid in shape, embedded shallowly in sternum and movable.

#### Color in life.

Carapace dorsally brown, beige, or pink; rostrum margins red to reddish brown; chelae olivaceous green to brown; dactyl and propodus tubercles cream or yellow; pereiopods white, cream, or yellowish gray, rarely light blue; abdomen terga dorsally brown or beige, bordered in crimson; ventral surfaces cream or white.

#### Specimens examined.

Cambarus carinirostris were collected from four counties at eight locations. Localities and demographics are listed below.

**BROOKE COUNTY:** Cross Creek at entrance to Bruin Drive adjacent to Brooke High School, 40.306442 -80.5997; 4 September 2005 – (WLU 05090401), 2 II♂. RT 2 crossing of nameless tributary 2.27 km (1.41 mi) S of Beech Bottom, 40.23163 N / 80.6523 W; 28 June 2005 – (WLU 05072801), 2 II♂. **HANCOCK COUNTY:** Hardin Run 0.81 km (0.5 mi) from CR 2-7/RT 2 intersection on CR 2-7, 40.533314 -80.60326; 23 August 2005 – (WLU 0508230), 1 I♂, 2 II♂, 2 ♀. **MARSHALL COUNTY:** Boggs Run at RT 2 crossing, 40.02481 N / 80.72577 W; 28 July 2005 – (WLU 05072801), 1 II♂, 1♀. Long Run at Long Run/Fish Creek confluence, 39.805878 -80.8052; 20 July 2005 – (WLU 05072002), 3 ♀. Nameless tributary at RT 2 crossing adjacent to Columbia Chemical operations, 39.85933 -80.79305; 28 July 2005 – (WLU 05072803), 6 I♂, 1 ♀. **WETZEL COUNTY:** Proctor Creek at RT 2 crossing, 39.70037 -80.81791; 9 July 2008 – (WLU 08070901), 5 II♂, 1 ♀. RT 2 crossing of nameless tributary at Marshall/Wetzel County line, 39.720192 -80.82281; 20 July 2005 – (WLU 05072001), 1 II♂.

#### Distribution.

**Figure 6.**
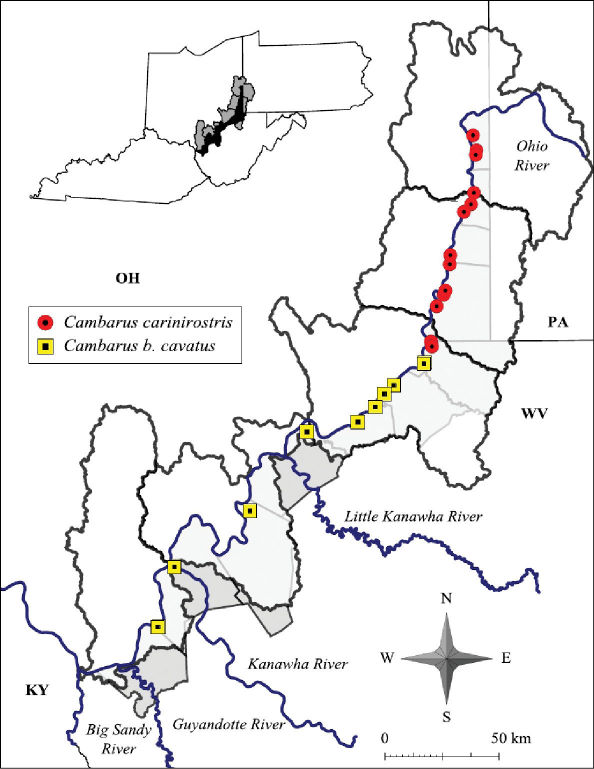
Cambarus carinirostris and Cambarus bartonii cavatus distribution along the West Virginia portion of the Ohio River floodplain

Cambarus carinirostris ranges from central West Virginia north through the Monongahela River system in West Virginia and Pennsylvania and the Allegheny River system in Pennsylvania and New York (Thoma and Jezerinac 1999).The western extent of Cambarus carinirostris is the Flushing escarpment in eastern Ohio (Thoma and Jezerinac 1999). Cambarus carinirostris were collected only from the northern basins along the floodplain, including Upper Ohio North, Upper Ohio South, and Middle Ohio North (Figure 6). Within the Middle Ohio North basin it was collected in the extreme northern regions of the basin. The southern limit of this species’ range in the floodplain is Proctor Creek, Wetzel County. Cambarus (Cambarus) bartonii cavatus Hay, 1902 replaces this species in Fishing Creek. The distribution of Cambarus carinirostris is the same as reported by Jezerinac et al. (1995).

#### Morphometrics.

Cambarus carinirostris is a moderate sized crayfish. Mean TCL was 29.1 mm (n = 29, SE = 5.61). The largest individual was a form I male with a TCL of 39.4 mm collected from Holbert Run in Hancock County. The largest female was also collected from Holbert Run, and had a TCL of 32.1 mm. Morphometric data for Cambarus carinirostris is presented in Table 2.

**Table 2.** West Virginia Ohio River Floodplain Cambarus carinirostris morphometrics

Sex	N	Minimum	Maximum	Mean	Standard Deviation
Male I					
Carapace Length	7	34.7	39.4	36.6	2.5
Palm Length	7	6.9	11.1	7.2	3.7
Areola Width	7	2.5	3.2	2.7	0.4
Areola Length	7	9.1	15.9	9.3	6.4
Male II					
Carapace Length	13	20.7	34.2	27.9	4.9
Palm Length	13	4.2	8.8	6.0	2.0
Areola Width	13	1.7	2.7	2.1	0.4
Areola Length	13	7.3	12.6	9.1	3.0
Female					
Carapace Length	9	20.4	32.1	26.7	4.4
Palm Length	9	4.5	7.3	5.6	1.0
Areola Width	9	1.8	2.3	2.1	0.3
Areola Length	9	7.1	11.7	8.3	2.0

#### Habitat and natural history.

**Figure 7.**
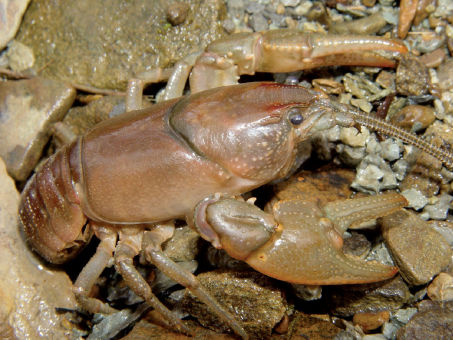
Cambarus carinirostris, Upper Ohio South basin, Ohio County, West Virginia – WLU 05072002

Cambarus carinirostris (Figure 7) inhabits lotic water bodies, with a preference for headwater streams (Jezerinac et al. 1995; Thoma and Jezerinac 1999). Most specimens collected along the floodplain were taken in first and second order streams. Within these environments, Cambarus carinirostris frequented spaces beneath slab boulders, large boulders, and various substrate debris. When the substrate permits, Cambarus carinirostris constructs burrow networks in the stream bank (Jezerinac et al. 1995; Loughman et al. 2009); however, no Cambarus carinirostris were collected from burrows in this study. Loughman et al. (2009) found that Cambarus carinirostris likely created the majority of stream bank burrows in northern West Virginia, given the scarcity of other burrowing species in northern portions of the floodplain.

Cambarus carinirostris also was collected from larger streams, where it inhabits side pools, eddies, and stream margins. The species appears to be limited to marginal habitats in larger ordered streams through competitive exclusion with larger, more aggressive species such as Orconectes obscurus (Hagen, 1870), and Cambarus robustus Girard, 1852, both of which were collected with Cambarus carinirostris. Seasonal data for Cambarus carinirostris are presented in Table 3.

**Table 3.** Seasonal data for West Virginia Ohio River Floodplain Cambarus and Fallicambarus species.

Species	J	F	M	A	M	Jn	J	A	S	O	N	D
Cambarus (Cambarus) carinirostris												
Male 1						×	×	×	×			
Male 2							×	×				
Females							×	×				
Ovigerous Females												
Cambarus (Cambarus) bartonii cavatus												
Male 1			×	×			×					
Male 2							×					
Females			×	×			×					
Ovigerous Females												
Cambarus (Procambarus) robustus												
Male 1				×	×		×			×		
Male 2				×	×				×	×		
Females					×		×			×		
Ovigerous Females												
Cambarus (Tubericambarus) thomai												
Male 1	×	×	×	×	×			×	×			
Male 2			×	×								
Females			×	×			×					
Ovigerous Females			×	×								
Fallicambarus (Cambarus) fodiens												
Male 1		×	×	×								
Male 2												
Females		×	×	×								
Ovigerous Females		×	×									

#### Conservation status within study area.

Cambarus carinirostris populations along the floodplain are stable and do not warrant special conservation action.

### Cambarus (Cambarus) bartonii cavatus

Hay, 1902

Cambarus bartonii cavatus Hay 1902:435. Faxon 1914:425. Taylor et al. 1996:29. Taylor et al. 2007.
Cambarus (Bartonius) bartonii Ortmann 1905b:120 [in part].
Cambarus (Cambarus) bartoni cavatus Ortmann 1931:127.
Cambarus (Cambarus) bartonii cavatus Fowler 1912:341. Hobbs, 1969:109, fig. 5; 1974:11, fig. 25; 1989:14, fig. 31. Jezerinac et al. 1995:84, fig. 39A–39H. Taylor and Schuster 2004:63, figs. 32, 33A–33H.

#### Diagnosis.

Rostrum broad; margins reduced, subparallel, terminating cephalically in a gentle angle to form acumen; anterior region of rostrum excavated; acumen consisting of a single upturned spiniform tubercle; postorbital ridges truncated, cephalic margin with weak tubercle; cephalothorax oval shaped and slightly dorsoventrally flattened in profile; 2–3 punctations across narrowest region of areola; branchiostegal region moderately punctate, with small tubercles; chelae broad and robust; mesial surface of palm consisting of two rows of defined tubercles; first row with 5–8 rounded tubercles; second with 3–4 tubercles; two prominent subpalmar tubercles present; first form gonopods contiguous at base, with 2 terminal elements bent 90° to the base; central projection with shallow subapical notch; total length of central projection equal to mesial process length; second form gonopod non-corneous and blunt; mesial process bulbous, truncating distally; annulus ventralis rhomboid in shape, embedded shallowly in sternum and movable.

#### Color in life.

Carapace dorsally olivaceous brown, beige, or tan; rostrum margins chestnut brown to brown; chelae olivaceous green to brown; dactyl and propodus denticles cream or yellow; pereiopods tan, light green, cream, or gray; abdomen terga dorsally brown or beige, bordered in gray; ventral surfaces cream or white.

#### Specimens examined.

Cambarus bartonii cavatus were collected from six counties at 15 locations. Collection locales and demographics are listed below.

**JACKSON COUNTY:** Flooded field adjacent to RT 33 S, 9.72 air km (6.04 mi) N of Ravenswood, 39.04274 -81.7827; 3 April 2005 – (WLU 05040301), 3♀. Little Sandy Creek, at intersection of RT 68/CR 8, 38.991497 -81.761765; 21 July 2006 – (WLU 06012103), 1 II *♂.* Vernal pool complex adjacent to railroad tracks 3.56 km (2.21 mi) N of Ravenswood, 39.09015 -81.79469; 3 April 2005 – (WLU 05040302), 1♀. West Creek, at intersection of CR 12 /CR 10, 38.924362 -81.94200; 20 July 2006 – (WLU 06072001), 1 II ♂, 1♀. **MASON COUNTY:** Pin oak swamp adjacent to Point Pleasant Moose Lodge in Wagner, 38.833603 -82.12227; 6 June 2005 – (WLU 05060601), 2 *♀.* Roadside ditch adjacent to RT 2, 9.17 km (5.7 mi) S of Point Pleasant, 38.80469 -82.18821; 4 March 2005 – (WLU 05030401), 2 I♂, 1 ♀. Roadside ditch adjacent to RT 2 N, 2.91 km (1.81 mi) N of Clover, 38.589428 -82.19548; 4 March 2005 – (WLU 05030402), 1 ♀. Roadside ditch adjacent to RT 2 N, 1.93 km (1.2 mi) N of Glenwood, 38.58816 -82.201004; 3 April 2005 – (WLU 05040305), 1 I♂, 2 ♀. Roadside ditch adjacent to RT 2 N 0.90 air km (0.56 mi.) N of Hogsett, 38.70056 -82.17708; 17 March 2005 – (WLU 05031707), 1 ♀. **PLEASANTS COUNTY:** Middle Island Creek at RT 2 crossing, 39.40328 -81.197624; 21 July 2006 – (WLU 06072104), 1 ♀. **TYLER COUNTY:** Nameless tributary at RT 2 crossing in Friendly, 39.513386 -81.06285; 28 July 2005 – (WLU 05072806), 1 I♂, 1 ♀. **WETZEL COUNTY:** Doolins Run at RT 2 crossing, 39.639576 -80.85607; 28 July 2005 – (WLU 05072805), 1 II♂. **WOOD COUNTY:** Big Run at CR 21-1 crossing, 39.364048 -81.45656; 21 July 2004 – (WLU 06072101), 1 ♀. Boaz Swamp Wildlife Management Area, 39.462868 -81.10855; 12 April 2004 – (WLU 04042101), 1 ♀. Nameless tributary crossing 3.54 km (2.2 mi) S of Parkersburg, 39.05142 -81.742836; 21 July 2006 – (WLU 06072106) 1 II♂, 1 ♀.

#### Distribution.

Cambarus bartonii cavatus ranges from northern Georgia and Tennessee through eastern Kentucky, east central Ohio and western Virginia (Taylor and Schuster 2004)*.* In West Virginia Cambarus bartonii cavatus is prevalent throughout basins associated with the lower reaches of the Kanawha system west of Kanawha Falls and basins draining into the Big Sandy River system. Cambarus bartonii cavatus floodplain populations inhabit the Middle Ohio North, Middle Ohio South, and Lower Ohio basins, and are the dominant secondary burrowing species inhabiting the floodplain (Figure 6). It is replaced in the Middle Ohio North basin in Proctor Creek with Cambarus carinirostris. A specimen collected from Doolin Run, Wetzel County, a tributary to Fishing Creek, represents the northernmost collection of this species in West Virginia. The distribution of this species has not changed since Jezerinac et al.’s (1995) survey in the late 1980’s.

#### Morphometrics.

Cambarus bartonii cavatus is a medium to large crayfish. The largest individual collected was a female with a 51.6 mm TCL taken from an ephemeral pool complex 3.6 km north of Ravenswood, Jackson County. The largest male collected was a form I collected from a roadside ditch 1.9 km north of Glenwood, Mason County, with a TCL of 45.6 mm. Mean Cambarus bartonii cavatus carapace length was 34.4 mm (n = 25, SE = 12.42) . Morphometric data for Cambarus bartonii cavatus is presented in Table 4.

**Table 4.** West Virginia Ohio River floodplain Cambarus bartonii cavatus morphometrics.

Sex	N	Minimum	Maximum	Mean	Standard Deviation
Male I					
Carapace Length	5	9.2	45.6	35.4	14.6
Palm Length	5	3.1	14.9	9.0	4.3
Areola Width	5	1.1	3.7	2.4	0.9
Areola Length	5	6.4	12.7	9.8	2.9
Male II					
Carapace Length	3	6.2	32.9	19.4	11.5
Palm Length	3	3.4	6.9	4.8	1.5
Areola Width	3	1.4	1.8	1.5	0.0
Areola Length	3	5.0	13.4	8.3	3.8
Female					
Carapace Length	17	18.8	51.6	39.0	9.1
Palm Length	17	3.5	11.1	8.9	3.9
Areola Width	17	2.2	2.5	2.2	0.7
Areola Length	17	7.5	3.9	10.4	2.5

#### Habitat and natural history.

**Figure 8.**
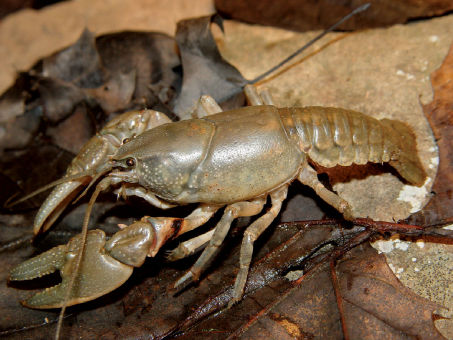
Cambarus bartonii cavatus, Lower Ohio basin, Cabell County, West Virginia– WLU 05031707

**Figure 9.**
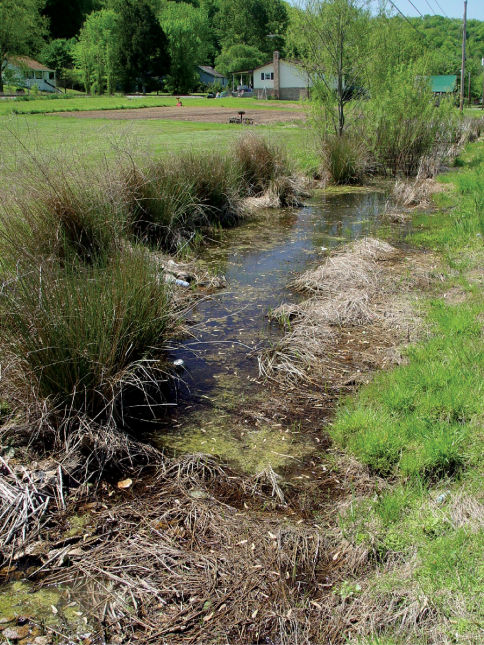
Glenwood, Mason County – Roadside Ditch Cambarus bartonii cavatus and Cambarus thomai utilized roadside ditches readily; these habitats proved important for floodplain crayfish populations. Additional species observed utilizing roadside ditches included Fallicambarus fodiens and Procambarus acutus acutus.

Cambarus bartonii cavatus (Figure 8) is a secondary burrowing species like Cambarus carinirostris, (Jezerinac et al. 1995). Along the floodplain, it utilized first and second order stream habitats, ephemeral wetlands, and roadside ditches (Figure 9). The species demonstrated a preference for roadside ditches, with 42.1% of individuals taken from this habitat. Ditches with an associated first-order stream produced particularly robust populations.

Within lotic systems, Cambarus bartonii cavatus prefers first through third order streams to larger streams. It burrows extensively in stream banks, particularly those composed of hardpan and similar regoliths. Burrows are intricate, with central shafts ranging in depth from 0.3 m to 1.0 m. At the terminus of the central shaft, enlarged chambers were always present with several branching auxiliary tunnels. One marked difference between Cambarus bartonii cavatus burrows and those of other burrowing species is the width of the central shaft and the dimensions of the central resting chamber.

The central shaft and central chamber of other floodplain burrowing species (e.g., Cambarus thomai Jezerinac, 1993, Fallicambarus fodiens (Cottle, 1863), were the width of the crayfish’s carapace at the widest point. Cambarus bartonii cavatus burrows did not follow this same pattern and, usually, were wide and oblong. Anecdotally, Cambarus bartonii cavatus burrows were readily identified by the presence of these structural components, but this method of identification was not used to definitively verify Cambarus bartonii cavatus presence at a site.

In late winter females comprised 66% of trap captures. Those captured in late winter/early spring all possessed active glair glands. This condition has been used in previous studies to indicate future egg extrusion and is likely the explanation for this increase in female activity (Hobbs 1981). No ovigerous females were collected during this study. Jezerinac et al. (1995) reported ovigerous specimens in West Virginia in July. A female retained in captivity collected in a roadside ditch 2.91 km north of Clover extruded eggs on 18 May 2005. Males captured in late winter/early spring did not show any trend toward any single demographic group, with an equal number of form I and form II individuals captured. Crayfish associates collected with Cambarus bartonii cavatus included Cambarus robustus, Cambarus thomai, Fallicambarus fodiens, Orconectes obscurus, Orconectes sanbornii (Faxon, 1884), Orconectes virilis and Procambarus acutus. Seasonal data for Cambarus bartonii cavatus are presented in Table 3.

#### Conservation status within study area.

Cambarus bartonii cavatus populations along the floodplain are stable and do not warrant special attention.

### Cambarus (Puncticambarus) robustus

Girard, 1852

Cambarus robustus  Girard 1852:90. Hagen 1870:80, fig. 167. Crocker and Barr 1968:118, figs. 30, 39, 48, 55, 65, 67, 81. Page 1985:439–440, figs 171–173. Taylor et al. 1996:29. Taylor et al. 2007:383.
Cambarus Bartonii robustus Faxon 1885:9.
Cambarus bartonii robustus Faxon 1890:622.
Cambarus (Bartonius) bartoni robustus Ortmann 1905b:122.
Cambarus (Cambarus) bartonii robustus Fowler 1912:341.
Cambarus (Cambarus) bartonii robustus Ortmann 1931:126.
Cambarus (Bartonius) robustus Creaser 1931:260.
Cambarus (Puncticambarus) robustus Hobbs 1969: 101, figs. 1c, 13a, 17o; 1974:21, fig. 77; 1989:27, fig. 104. Jezerinac et al. 1995:155–171, fig. 75a–75H. Taylor and Schuster 2004:103–106, figs. 74A, 74B, 75A–75H.

#### Diagnosis.

Rostrum narrow to slightly broad, margins reduced and parallel, terminating in gentle angle cephalically to form acumen terminating in a single upturned spiniform tubercle; postorbital ridges prominent, cephalic margin with tubercle; cephalothorax dorsoventrally flattened in profile, anterior portion weakly vaulted; 2–5 punctations across narrowest region of areola; branchiostegal region moderately punctate, with small tubercles; chelae robust; mesial surface of palm consisting of two rows of defined tubercles; first row with 7–9 rounded tubercles; second with 5–7 smaller tubercles; additional tubercles scattered over dorsal region of palm; three prominent subpalmar tubercles present; first form gonopods contiguous at base, with 2 terminal elements bent 90° to base; central projection with distinct subapical notch; total length of central projection equal to mesial process length; mesial process bulbous, truncating distally; second form gonopod non-corneous and and blunt; annulus ventralis rhomboid in shape, embedded shallowly in sternum and movable.

#### Color in life.

Carapace dorsally brown; cephalic region reddish brown, branchial region pinkish brown to light brown; cervical groove black; rostrum margins orange or red; chelae olivaceous green to green; tubercles on chelae yellow or orange; dactyl and fixed finger denticles cream or yellow; perieopods green or light blue; abdomen terga bodies dorsally brown or olivaceous brown; bordered in red, ventral surfaces cream or white.

#### Specimens examined.

Cambarus robustus were collected from five counties at seven localities, as listed below.

**HANCOCK COUNTY:** Kings Creek at RT 2 crossing, 40.435715 -80.592514; 17 October 2005 – (WLU 05101701), 4 I♂, 2 II♂, 1 ♀. **MARSHALL COUNTY:** Long Run at Long Run/Fish Creek confluence, 39.805878 -80.8052; 30 October 2005 – (WLU 05103002), 2 *♀.* Fish Creek at RT 2 crossing, 39.808643 -80.81616; 30 October 2005 – (WLU 05103002), 1 I♂, 2 ♀. **PLEASANTS COUNTY:** Ben’s Run at RT 2 crossing, 39.46337 -81.08457; 28 July 2005 – (WLU 05072802), 3 ♀. **TYLER COUNTY:** Cow House Run at RT 2 crossing, 39.551327 -81.01001; 28 July 2005 – (WLU 05072804), 1 II♂. **WETZEL COUNTY:** Fishing Creek at RT 2 crossing, 39.63576 N/ -80.85848 W; 20 July 2005 – (WLU 05072001), 1 I♂, 1 ♀. Proctor Creek at RT 2 crossing, 39.70037 N/ -80.81791 W; 9 July 2008 – (WLU 08070901), 2 II♂, 1 ♀.

#### Distribution.

**Figure 10.**
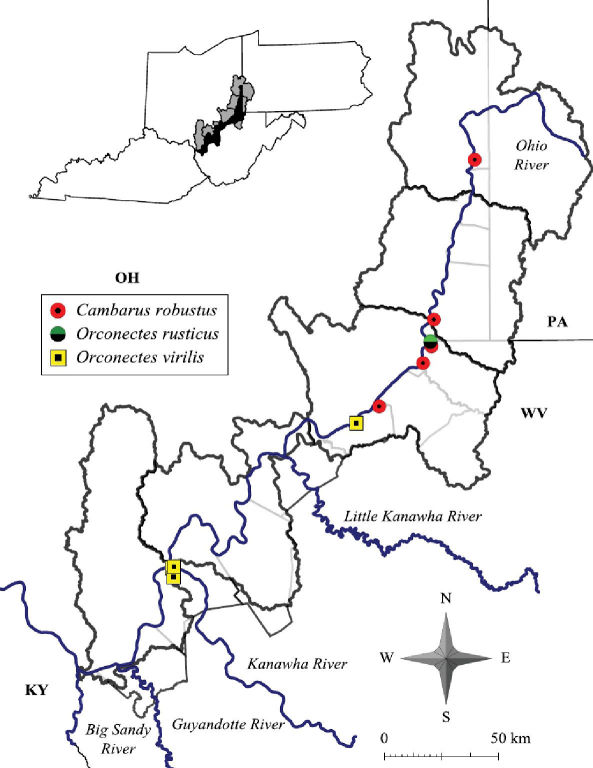
Cambarus robustus, Orconectes rusticus and Orconectes virilis distribution along the West Virginia portion of the Ohio River floodplain.

Cambarus robustus has an extensive distribution, ranging from southern Ontario and central New York south to North Carolina and Virginia, and west to Illinois (Taylor and Schuster 2004).Given thisextensive range, Cambarus robustus likely represents a species complex.Floodplain Cambarus *robustus* were collected from the Upper Ohio North and Middle Ohio North basins (Figure 10), but has also been collected in the Middle Ohio South and Lower Ohio drainages outside the floodplain (Z. J. Loughman, unpublished data). Jezerinac et al. (1995) collected Cambarus robustus from Harman Creek, Brooke County, in the northern panhandle. The only other northern panhandle location where the species was collected in was the Fish Creek system in Marshall County. Loughman et al. (2009) commented on this disjunct distribution and hypothesized that it could be the result of separate postglacial invasions. Most Cambarus robustus populations present along the floodplain occur in the Middle Ohio North basin (60% of streams).

Cambarus robustus was likely under surveyed during this effort. This species prefers free-flowing streams more similar to mainstem rivers rather than habitats associated with big river confluences like those sampled in this survey.

#### Morphometrics.

The largest individual collected was a 46.6 mm TCL form II male collected in Kings Creek, Hancock County. The largest female was also taken there and was 36.0 mm TCL. Mean Cambarus robustus TCL was 33.0 mm (n = 20, SE = 5.5). Morphometric data are presented in Table 5.

**Table 5.** West Virginia Ohio River floodplain Cambarus robustus morphometrics.

Sex	N	Minimum	Maximum	Mean	Standard Deviation
Male I					
Carapace length	6	30.7	46.4	35.0	8.4
Palm Length	6	6.5	13.1	8.8	3.4
Areola Width	6	1.8	2.7	2.1	0.7
Areola Length	6	10.4	16.8	9.4	4.34
Male II					
Carapace length	5	34.9	46.6	37.3	8.3
Palm Length	5	3.6	13.7	9.6	3.1
Areola Width	5	0.6	3.8	2.4	1.1
Areola Length	5	5.5	18.0	12.6	4.6
Female					
Carapace length	10	13.8	36.0	21.0	10.8
Palm Length	10	2.7	8.3	4.2	3.5
Areola Width	10	0.5	7.2	7.5	7.3
Areola Length	10	4.7	7.3	2.9	4.8

#### Habitat and natural history.

**Figure 11.**
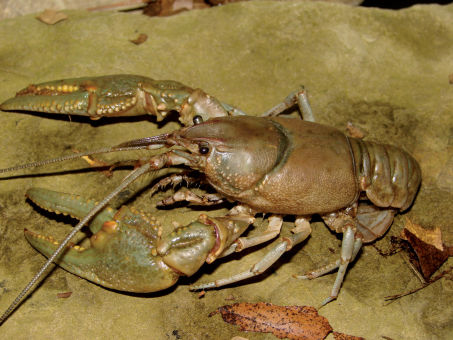
Cambarus robustus, Upper Ohio North basin, Hancock County, West Virginia – WLU 05101701

Cambarus robustus (Figure 11) inhabits 3_rd_ through 5_th_ ordered streams that dissect the floodplain. Preferred microhabitats included leaf packs, boulder fields, and spaces beneath large slab boulders. Cambarus robustus observed in Ben’s Run burrowed into hardpan substrates of pools, and readily used available leaf packs. Many individuals eluded capture in this stream, but were observed resting at the entrances to these burrows. No Cambarus robustus were collected from headwater streams in this effort. Crayfish associates included Cambarus carinirostris, Cambarus bartonii cavatus, and Orconectes obscurus. Seasonal data for Cambarus robustus are presented in Table 3.

#### Conservation status within study area.

Cambarus robustus populations along the floodplain are stable and do not warrant special attention.

### Cambarus (Tubericambarus) thomai

Jezerinac, 1993

Cambarus diogenes  Girard 1852:88 [in part]. Williamson 1899:48 [in part]. Ortmann 1905:398 [in part]. Newcombe 1929:286. Rhoades 1944a:146 [in part]; 1944b:98 [in part].
Cambarus diogenes diogenes Hay 1899:959 [in part]. Marlow 1960:233.
Cambarus (Bartonius) diogenes Ortmann 1906:402. Turner 1926:168 [in part].
Cambarus (Lacunicambarus) diogenes diogenes Hobbs 1969: 110 [in part]. Bouchard 1972:56 [in part], 1975:595 [in part]. Lawton 1979:47. Thoma and Jezerinac 1982:136. Jezerinac and Thoma 1984:123. Jezerinac 1985:7.
Cambarus (Lacunicambarus) diogenes Jezerinac 1985:7. Hobbs 1989: 24, fig. 88.
Cambarus (Tubericambarus) thomai Jezerinac 1993:532, fig. 4. Jezerinac et al. 1995:172–179, fig. 84a–84h. Taylor and Schuster 2004:121–123, figs. 92, 93A–93H. Loughman 2010:46-50, fig. 12.
Cambarus thomai Taylor et al. 1996:29. Taylor et al. 2007:383.

#### Diagnosis.

Rostrum slightly broad, margins converging to form acumen terminating in single reduced, upturned tubercle; postorbital ridges reduced, rarely terminating in small tubercle; cephalothorax dorsolaterally compressed in profile and vaulted; areola obliterated; branchiostegal region devoid of tubercles; chelae robust and diamond shaped; mesial surface of palm with disorganized prominent tubercles, mesialmost tubercles serrate; basiodactyl row consisting of 5–9 reduced rounded tubercles; first form male gonopods contiguous, with 2 terminal elements bent 90° to the shaft; central projection truncated distally and lacking sub-apical notch; total length of central projection equal to mesial process length; mesial process short, truncating distally; second form gonopod non-corneous and blunt; annulus ventralis rhomboid in shape with deep “S” shaped sinus, embedded shallowly in sternum, and movable.

#### Color in life.

Carapace dorsally brown, light green, olive, light blue, or blue grey; rostrum margins orange or red; chelae body light green, light brown, or blue; propodus light blue or light green; dactyl and propodus denticles cream or yellow; pereiopods tan, light green, cream, or gray; abdomen body light green, light blue gray or brown; tubercles covering chelae light yellow, cream, or orange; two light dorsal stripes present on dorsal surface of abdomen; ventral surface cream or white.

#### Specimens examined.

Cambarus thomai were collected from eight counties at 26 localities, listed below.

**CABELL COUNTY:** Green Bottom Swamp at Hoeft Marsh Wildlife Management Area, 38.58616 N / 82.24878 W; 2 April 2009 – (WLU 09040201), 2 I♂. **HANCOCK COUNTY:** Tomlinson Run backwater at RT 2 crossing, 40.54026 -80.628075; 30 March 2006 – (WLU 06033001), 3 I♂. **JACKSON COUNTY:** Flooded field adjacent to RT 33 S, 9.72 air km (6.04 mi) N of Ravenswood, 39.04274 -81.7827; 18 March 2005 – (WLU 05031803), 7 I♂, 3 O♀. Vernal pool complex adjacent to railroad tracks 3.56 km (2.21 mi) N of Ravenswood, 39.09015 -81.79469; 3 April 2005 – (WLU 05040302), 3 I♂, 1 II♂. **MASON COUNTY:** Ditch adjacent to RT 2 N, 1.77 km (1.10 mi) N of Rt 2 railroad crossing in Ashton, 38.63165 -82.16464; 18 March 2005 – (WLU 05031805), 3 I♂, 1 II♂. Krodel Park marsh adjacent to Fort Randolph reproduction, 38.785404 -82.12209; 5 March 2005 – (WLU 05030502), 4 I♂. Maple swamp adjacent to RT 2 railroad crossing in Ashton, 38.622005 -82.16758; 26 March 2004 – (WLU 04032601), 18 I♂, 2 ♀; 30 March 2004 – (WLU 04033001), 6 I♂, 3 O♀, 1 ♀; 28 April 2004 – (WLU 04042801), 4 I♂,1 O♀, 2 ♀; 18 March 2005 – (WLU 05031805), 2 I♂. Pin oak swamp adjacent to Point Pleasant Moose Lodge in Wagner, 38.833603 -82.12227; 26 March 2004 – (WLU 04032601), 3 I♂; 12 April 2004 – (WLU 04041205), 3 I♂, 2 O♀. Red-Osier Dogwood swamp adjacent to RT 33 S in Hartford, 39.008915 -81.99847; 5 March 2005 – (WLU 05030504), 1 I♂. Roadside ditch adjacent to RT 2 N, 2.91 km (1.81 mi) N of Clover, 38.589428 -82.19548; 4 March 2005 – (WLU 05030402), 3 I♂. Roadside ditch adjacent to RT 2, 9.17 km (5.7 mi) S of Point Pleasant, 38.80469 -82.18821; 12 April 2004 – (WLU 04041206), 6 I♂. Roadside ditch adjacent to RT 2 N, 0.22 air km (0.14 mi) N of Hogsett, 38.694496 -82.1765; 17 March 2005 – (WLU 05031707), 5 I♂. Roadside ditch adjacent to RT 2 N 0.90 air km (0.56 mi.) N of Hogsett, 38.70056 -82.17708; 17 March 2005 – (WLU 05031707), 4 I♂, 1 II♂. Roadside ditch adjacent to RT 2 N 1.93 km (1.2 mi) N of Glenwood, 38.58816 -82.201004; 3 April 2005 – (WLU 05040305), 1 I♂. Roadside ditch adjacent to RT 62 N, 0.34 km (0.21 mi) N of Hallwood, 38.97562 -82.081314; 17 March 2005 – (WLU 05031706), 1 I♂. Roadside ditch adjacent to RT 62 S at Mountaineer Power Plant, 38.974934 -81.94418; 5 March 2009 – (WLU 05030509), 2 I♂. Slough adjacent to RT 33 N in Mason, 39.00983 -82.03899; 5 March 2005 – (WLU 05030505), 1 I♂; 17 March 2005 – (WLU 05031704), 6 I♂, 4 ♀. Vernal pool complex at RT 2/Lighthouse Gospel Church Road intersection, 38.82201 -82.13136; 17 March 2005 – (WLU 05031707), 2 I♂, 2 O♀. **PLEASANTS COUNTY:** Ohio River embayment 4.03 air km (2.52 mi) S of St. Mary’s, 39.397575 -81.202415; 12 April 2004 – (WLU 05030506), 1 II♂, 1 OF. Vernal pool adjacent to RT 2 N across from Cytec Community Fishing Area, 39.347824 -81.32024; 5 March 2005 – (WLU 04041203), 2 I♂. **TYLER COUNTY:** Maple swamp adjacent to RT 2 S in Friendly, 39.50822 -81.06736; 20 March 2004 – (WLU 04032001), 2 I♂. **WETZEL COUNTY:** Maple Swamp adjacent to RT 2 S in New Martinsville, 39.32582 -80.866234; 2 April 2004 – (WLU 04040201), 8 I♂, 2 ♀; 21 March 2006 – (WLU 06032104), 3 I♂, 1 II♂. (23.) Ohio River backwater at Marshall/Wetzel County line, 39.717846 -80.514959; 2 April 2004 – (WLU 04040203), 2 I♂; 21 March 2006 – (WLU 06032101), 3 I♂. **WOOD COUNTY:** Bellville Wildlife Management Area 4.03 km (2.50 mi) S of Bellville, 39.132915 -81.730865; 5 March 2005 - (WLU 05030507), 6 I♂. Boaz Swamp Wildlife Management Area, 39.462868 -81.10855; 25 March 2004 – (WLU 04032501), 1 I♂; 12 April 2004 – (WLU 04041203), 2 I♂, 1 O♀; 5 March 2005 – (WLU 05030509), 1 I♂. Lee Creek at CR 11 crossing, 39.153275 -81.73507; 2 April 2004 – (WLU 04040203), 2 I♂.

#### Morphometrics.

Cambarus thomai is the largest burrowing crayfish occurring in West Virginia, and the most frequently collected species in this study. The largest individual collected was a form I male, 53.6 mm TCL from Bellville, Wood County. The largest female measured 38.6 mm TCL and was collected from a flooded field 1.1 km north of Ravenswood, Jackson County. Mean Cambarus thomai carapace length was 37.0 mm (n = 148, SE = 5.41). Morphometric data for Cambarus thomai are presented in Table 6.

**Table 6.** West Virginia Ohio River floodplain Cambarus thomai morphometrics.

Sex	N	Minimum	Maximum	Mean	Standard Deviation
Male I					
Carapace length	119	26.4	53.6	37.3	5.7
Palm Length	119	5.7	34.7	17.2	8.8
Areola Width	—	—	—	—	—
Areola Length	119	3.4	18.3	8.3	2.84
Male II					
Carapace length	5	22.3	46.1	30.5	8.0
Palm Length	5	5.2	25.6	16.8	7.0
Areola Width	—	—	—	—	—
Areola Length	5	5.1	9.6	7.7	1.5
Female					
Carapace length	24	19.2	38.6	30.6	5.4
Palm Length	24	5.5	25.6	15.8	7.5
Areola Width	—	—	—	—	—
Areola Length	24	3.2	17.9	7.3	3.7

#### Distribution.

Cambarus thomai distribution includes western Pennsylvania, central and eastern Ohio, central and western West Virginia and eastern Kentucky (Taylor and Schuster 2004). (Ortmann (1905a, 1906) was the first to mention the presence of Cambarus thomai (= Cambarus diogenes Girard, 1852) in Brooke and Hancock counties, stating that populations persisting in both counties were stable. Newcombe (1929) documented the species in Hancock and Brooke counties, and like Ortmann, identified the species as Cambarus diogenes. Jezerinac described Cambarus thomai in 1993 based on material from West Virginia in his description (Jezerinac 1993), but. questioned the validity of Newcombe’s records. Jezerinac (1993) found northern Cambarus thomai populations problematic, specifically those occurring in Brooke County. This study did not collect any specimens from Brooke County, but specimens were collected in Tomlinson Run Backwater, validating previous records for Hancock County. Cambarus thomai was not taken in Brooke County during this study, but has been collected recently from portions of the county not associated with the floodplain.

**Figure 12.**
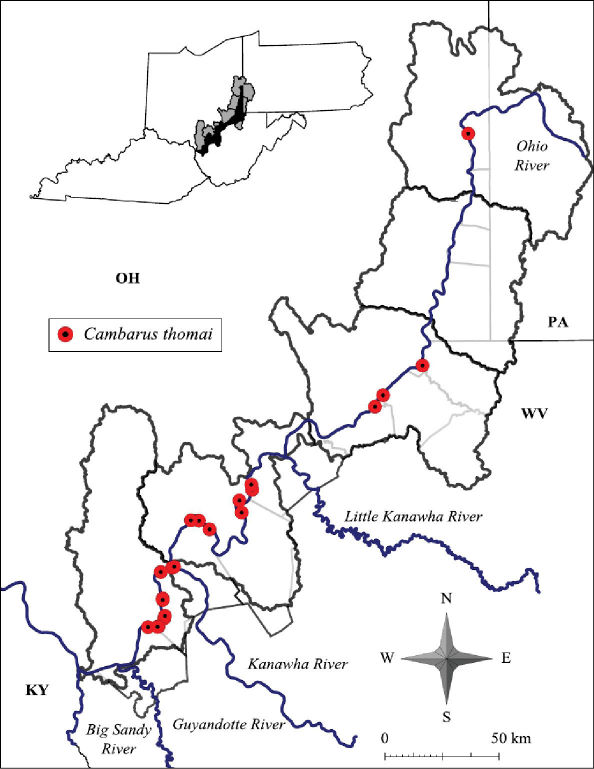
Cambarus thomai distribution along the West Virginia portion of the Ohio River floodplain

Cambarus thomai was collected from the Upper Ohio North, Middle Ohio North, Middle Ohio South, and Lower Ohio basins (Figure 12). Specimens from Jackson County, Middle Ohio South basin, represent county records. It is absent from the Upper Ohio South basin and occurs again in the Upper Ohio North basin (Figure 12). Within the Upper Ohio North, Cambarus thomai was collected, but not in large numbers. Cambarus thomai populations enter the Upper Ohio North basin from the Tuscarawas River in Eastern Ohio. Different soil types are found in the Upper Ohio North and South basins, which could explain the species’ distribution. Another possibility controlling Cambarus thomai distribution is the increased agricultural land use practices and declining riparian habitat that has sharply increased in the Upper Ohio South and North basins.

In the Middle Ohio North, Middle Ohio South, and Lower Ohio basins, Cambarus thomai is stable. Mason County contains substantial Cambarus thomai populations, with the species documented at every site (n = 18) sampled in the county. Populations decline north of these basins. The most substantial northern population occurs in New Martinsville, Wetzel County. Ortmann (1906) commented on this population based on surveys in the late 1800’s, noting how numerous burrows were in “bottomlands” adjacent to the Ohio River.

#### Habitat and natural history.

**Figure 13.**
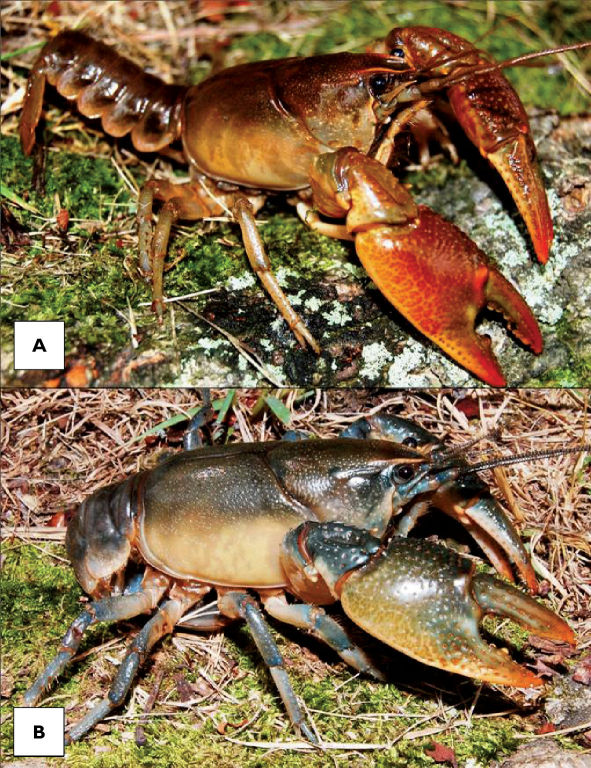
Cambarus thomai, Middle Ohio South basin, Mason County. Amber – WLU 04032601 (A.) and blue–green - WLU 04032605 (B.) colorphases. This species is the most prevalent crayfish along the Ohio River floodplain and constructs intricate burrows in lentic habitats in the Upper Ohio South, Middle Ohio North and South, and Lower Ohio basins.

Cambarus thomai (Figure 13) was the most frequently collected burrowing crayfish along the Ohio River floodplain. Marshes, swamps, embayments, wet fields, ephemeral pools, ponds, roadside ditches, and bottomland forests are habitats utilized by Cambarus thomai. Population density appears to be directly correlated with mature forest canopies, with a preference for ephemeral pool systems, bottomland forests, and marsh habitats.

Population densities decline in exposed agricultural fields. The species responds negatively to livestock even when adequate habitat is available. These pasture habitats exhibit soil compaction, excess nutrients, and low browse lines. A lack of vegetation possibly expedites drawdown conditions with increased levels of evapotranspiration. Exposed conditions and frequent manipulation of topsoil appear to limit Cambarus thomai density in agricultural settings.

**Figure 14.**
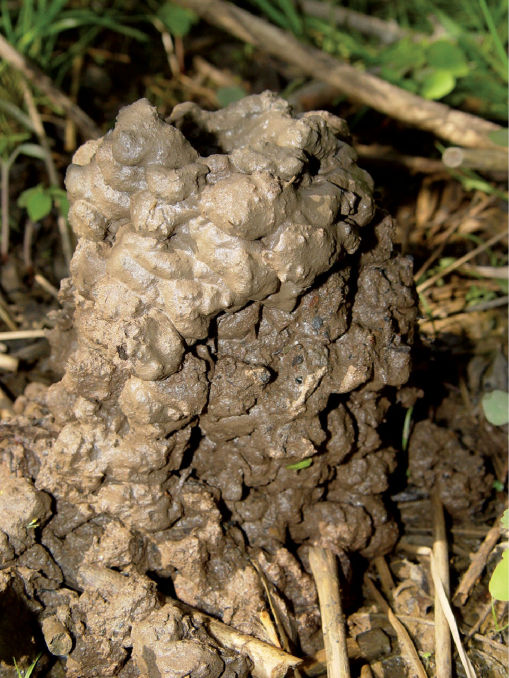
Cambarus thomai chimney, Middle Ohio North basin, Wetzel County, West Virginia. Cambarus thomai chimneys were numerous throughout the southern regions of the floodplain. The chimney pictured measured 18 cm in height and 10 cm in diameter.

Cambarus thomai uses surface waters during late-winter and early-spring. During all other seasons it was collected from burrows, which are complex, with a 0.3 m to 1.5 m deep central shaft ending in a resting chamber. Central shafts often have multiple ancillary tunnels prior to the resting chamber. Resting chambers also possess additional tunnels, particularly from their floors. Vegetation was frequently found in these auxiliary tunnels. In many instances a short 10–20 cm central shaft bifurcates into two complete central shafts, each ending in its own central chamber. Chimneys often were associated with these burrows (Figure 14).

Cambarus thomai were nocturnal, and displayed stylized behaviors while resting in their burrow portals. They rest with their antennae held laterally and their chelae barely breaching the burrow’s entrance. If pressure pulses are sent through the soil, they orient their antennae toward the pulse without shifting body position. If pulses continued, crayfish either retreated into their burrows or left their burrow’s to investigate the pulse source. The majority of Cambarus thomai observations at burrow portals occurred in June and July. During late winter and early spring, several form I males and ovigerous females were observed nocturnally cruising and feeding on periphyton in ephemeral pools.

**Figure 15.**
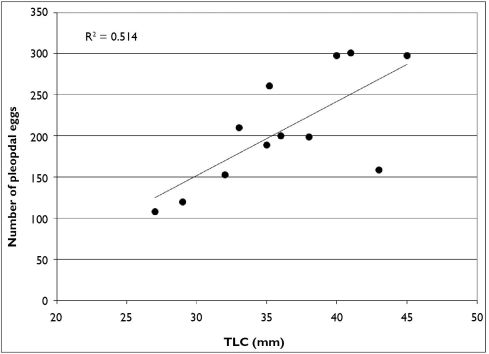
Relationship between carapace length and pleopodal egg number in Cambarus thomai

As stated previously, February through April, Cambarus thomai uses surface waters extensively. Eighty-six percent of trap captures were form I males. Ovigerous females (n = 12) also used surface waters, with 50% of females captured at this time carrying eggs. Linear regression analysis of ovigerous females indicates there is not a strong relationship between carapace length and the number of pleopodal eggs (Figure 15). Egg counts ranged from 108–304 eggs per female. Pleopodal egg diameter ranged from 1.51–2.47 mm, with a mean diameter of 2.09 mm.

Given the high percentage of ovigerous females captured in late winter and early spring, mating likely occurs in the fall. Females carry sperm throughout the winter and extrude eggs in early spring when ephemeral pool hydroperiods are at their most active. Instars are carried by females throughout the spring, and released at the beginning of the summer season. This life history strategy enables neonates to mature throughout the summer and enter their first winter as juveniles. Jezerinac et al. (1995) collected ovigerous females in March, April, May, and June in West Virginia, and Taylor and Schuster (2004) collected a single ovigerous female in Kentucky in March. Our results validated previous seasonal data for Cambarus thomai as presented in Table 3.

Cambarus thomai neonates used surface waters throughout the summer season (May–September) and were the only demographic observed at this time. Dip netting yielded large numbers of young-of-the-year in July and August; however, whether neonates remain in surface waters may depend on water availablility throughout the fall into winter. During drawdown in several sites in Mason County, juveniles were observed burrowing.

Neonate utilization of surface waters may be a dispersal mechanism to enable colonization and equally distribute individuals throughout wetlands or redistribute individuals into areas of high productivity. Nocturnal searches found Cambarus thomai utilizing surface waters rather than relying on burrows. On several occasions individuals would seek cover under substrate debris in surface waters although burrows were readily available.

#### Conservation status within study area.

Cambarus thomai populations are stable within the Middle Ohio North, Middle Ohio South and Lower Ohio basin. Additional survey efforts are needed in the Upper Ohio North and Upper Ohio South basins to determine the status of northern populations.

### Fallicambarus (Creaserinus) fodiens

(Cottle, 1863)

Astacus fodiens Cottle 1863:217.
Cambarus argillicola Faxon 1884:115.
Cambarus (Bartonius) argillicola Ortmann 1905b:120.
Bartonius argillicola  Wiliamson 1907:749.
Cambarus (Cambarus) fodiens Fowler 1912:341.
Cambarus fodiens Huntsman 1915:158, figs. 8f, 9d, 10e, 11a, 12e. Crocker and Barr 1968:129–135, figs. 28, 37, 46, 57, 62, 85.
Cambarus (Bartonius) fodiens Creaser 1931:260, fig. 37.
Fallicambarus (Creaserinus) fodiens Hobbs 1969: 111, fig. 20e; 1972:102, figs 83c, 84b, 85b; 1974:23, fig. 82; 1989:29, fig. 116. Page 1985:422, figs. 155–158. Hobbs and Jass 1988:39–43, figs.30a–30n. Jezerinac et al. 1995:180–187, figs. 88a–88h. Taylor and Schuster 2004:131–133, figs. 100, 101A–101H.
Fallicambarus fodiens Pflieger 1996:66–70, figs, 8A–8H. Taylor et al. 1996:30. Taylor et al. 2007:383.

#### Diagnosis.

Rostrum slightly broad and moderately excavated, deflected ventrally; margins converging to form acumen cephalically with reduced upturned tubercle; postorbital ridge reduced, not terminating in tubercle; cephalothorax dorsolaterally compressed in profile and vaulted; areola obliterated; branchiostegal region devoid of tubercles; chelae diamond shaped; mesial surface of palm with 2 distinct rows of tubercles; dorsalmost row consisting of 6–9 serrate tubercles; second row consisting of 3–6 circular tubercles; basiodactyl row consisting of 5–7 punctations; opposable surface of dactyl with distinct basal notch; junction of dactyl and propodus setiferous; first form gonopods basally contiguous, with 2 terminal elements bent 90° to shaft; central projection of populations on the floodplain possessing distinct subapical notch; total length of central projection equal to mesial process length; mesial process bulbous, truncating distally; second form gonopod non-corneous and blunt; subapical notch absent in second form gonopod; annulus ventralis rhomboid in shape with deep S-shaped sinus and C-shaped fossa; embedded shallowly in sternum, and movable.

#### Color in life.

Carapace dorsally and laterally tan, brown, reddish brown, or gray; cephalic and branchial region mottled with black or deep grey spots; chelae tan, deep gray, or gray brown; tubercles on chelae cream or light gray; distal region of dactyl and propodus increasingly orange; perieopods green or light grey; abdomen grey or olivaceous brown, with 2 distinct dorsal stripes; ventral surfaces cream or white.

#### Specimens examined.

Fallicambarus fodiens were collected from two counties at three locations in the current study, as listed below.

**CABELL COUNTY:** Green Bottom Swamp at Hoeft Marsh Wildlife Management Area, 38.58616 -82.24878; 2 April 2009 – (WLU 09040201), 2 I♂, 2 II♂, 1 O♀, 4♀. **MASON COUNTY:** Vernal pool complex at RT 2/Lighthouse Gospel Church Road intersection, 38.82201 -82.13136; 17 March 2005 – (WLU 05031707), 1 I♂, 1 ♀; 28 March 2005 – (WLU 05032801), 1 I♂, 3 ♀. Pin oak swamp adjacent to Point Pleasant Moose Lodge in Wagner, 38.833603 -82.12227; 26 February 2004 – (WLU 04022601), 1 II♂, 1 O♀; 26, March 2004 – (WLU 04032601), 3 I♂, 2 ♀; 30 March 2004 – (WLU 04033001), 1 I♂; 12 April 2004 – (WLU 04041202), 1 I♂; 28 April 2004 – (WLU 04042801), 1 II♂, 2 ♀.

#### Distribution.

**Figure 16.**
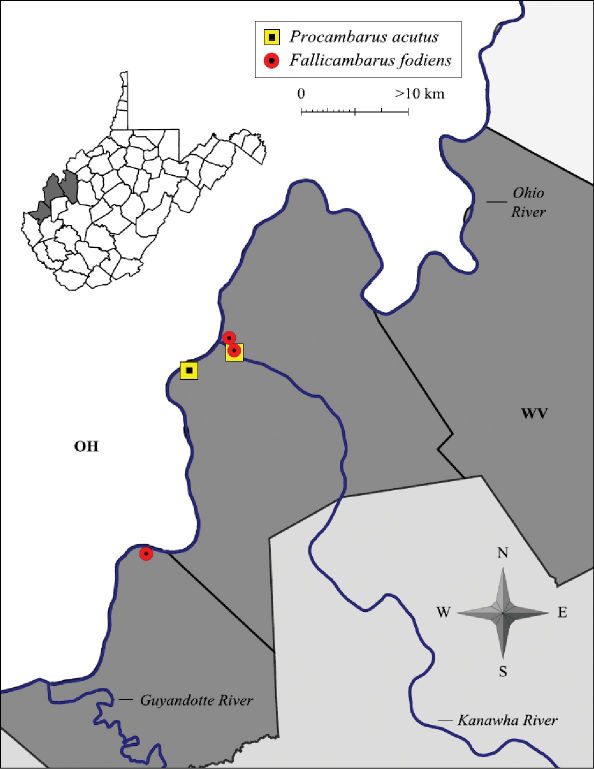
Fallicambarus fodiens and Procambarus acutus distribution along the West Virginia portion of the Ohio River floodplain

Fallicambarus fodiens is a wide-ranging species occurring along the Atlantic Slope in Maryland, Virginia, and North Carolina east to the Ohio and Mississippi river valleys excluding the majority of the Appalachian Mountains. Northern populations occur along the Great Lakes and in southern Ontario (Jezerinac et al. 1995). Fallicambarus fodiens were collected from the Lower Ohio Basin and Middle Ohio South Basin (Figure 16). Within the Middle Ohio South, all populations were within one km of the Middle Ohio South/Lower Kanawha basin border. Two previously unknown populations were discovered in Mason County including one adjacent to the Point Pleasant Moose Lodge in Wagner and another in Krodel Park, Point Pleasant. Increased efforts to find this species along the floodplain throughout the Middle Ohio South, Middle Ohio North and Lower Ohio basins were futile.

Jezerinac and Stocker (1987) theorized that Fallicambarus fodiens populations in West Virginia were Marietta River relicts. Currently, the Marietta River Valley is composed of the Kanawha River Valley. This hypothesis does help explain the scarcity of this species along the floodplain. Future survey efforts for Fallicambarus fodiens should focus on wetlands associated with the Kanawha River floodplain. The Moose Lodge wetland and its associated wetlands include that floodplain, which is 0.8 km from the Kanawha River and Ohio River confluence. The Moose Lodge wetland is composed of diverse bottomland forest with multiple ephemeral pools, which possess stable Fallicambarus fodiens populations. Habitat specialization may explain low Fallicambarus fodiens numbers elsewhere since little mature bottomland forests remain along the Ohio River floodplain. Apparently this habitat is needed for West Virginia’s disjunct population of Fallicambarus fodiens to persist.

#### Morphometrics.

Several animals were observed, but not disturbed due to the rarity of this species in West Virginia. The largest individual was a 40.1 mm TCL form I male collected at the Moose Lodge wetland, Mason County. The largest female collected was 37.3 mm TCL from an ephemeral pool complex located inside Krodel Park, Mason County. Mean carapace length for this species was 34.3 mm (n = 26, SE = 8.67). Morphometrics data for Fallicambarus fodiens is presented in Table 7.

**Table 7.** West Virginia Ohio River floodplain Fallicambarus fodiens morphometrics.

Sex	N	Minimum	Maximum	Mean	Standard Deviation
Male I					
Carapace Length	8	31.8	40.1	35.6	3.3
Palm Length	8	8.4	8.7	8.5	0.1
Areola Width	—	—	—	—	—
Areola Length	8	7.3	7.9	7.8	0.1
Male II					
Carapace Length	—	—	—	—	—
Palm Length	—	—	—	—	—
Areola Width	—	—	—	—	—
Areola Length	—	—	—	—	—
Female					
Carapace Length	14	31.6	37.3	33.5	6.4
Palm Length	14	6.1	21.9	15.0	7.4
Areola Width	—	—	—	—	—
Areola Length	14	5.3	10.1	7.7	2.1

**Figure 17.**
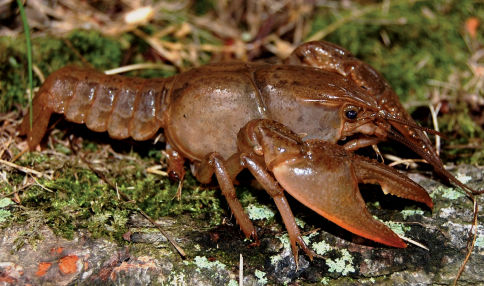
Fallicambarus fodiens, Middle Ohio South basin, Mason County, West Virginia - WLU 05031707

#### Habitat and natural history.

**Figure 18.**
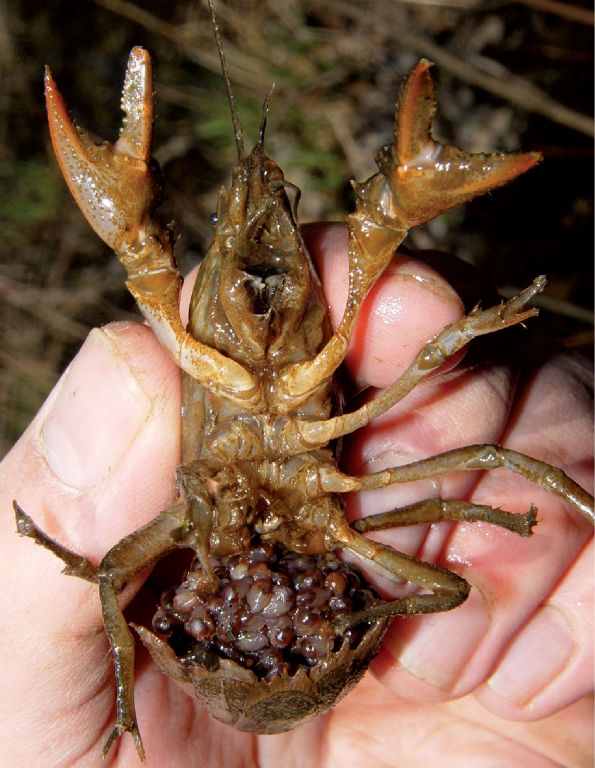
Ovigerous Fallicambarus fodiens, Middle Ohio South basin, Mason County, West Virginia. Ovigerous females were prevalent in surface waters from late February through early March. Females with pleopodal instars were observed from mid March through early May. The pictured specimen was released after capture.

Fallicambarus fodiens (Figure 18) in West Virginia is an ephemeral pool specialist. Within these ecosystems, colonies were associated with either lowland forest environments or open, wet fields. Similar habitat preferences for this species has been observed across its range (Guiasu 2007; Norrocky 1991; Taylor and Schuster, 2004). With the exception of the Greenbottom Swamp population, ephemeral systems are preferred over larger, more permanent water bodies. Fallicambarus fodiens colonies typically consist of 5–10 burrows for every 1 m2 of substrate. Within these colonies, the sex ratio of captured individuals was 1:1 male to female. Fallicambarus fodiens burrow morphology is simple, with the majority of excavated burrows consisting of a central shaft ranging in depth from 0.3 m to 1.0 m. One or two short ancillary tunnels often were present radiating from central shafts. These ancillary tunnels often are full of debris. Resting chambers usually were present at the terminus of these shafts, with either few or no ancillary tunnels radiating from them.

Several Fallicambarus fodiens females (n = 7) were collected carrying instars and eggs in February and March 2004 and 2005 (Figure 18). Egg extrusion along the floodplain occurred throughout the months of February and March. By early April, females were carrying first stage instars, with 4_th_ stage instars observed by late April. Jezerinac et al. (1995) also observed ovigerous females in February and March in West Virginia. Ovigerous females have been collected February through April in Illinois (Page 1985), Kentucky (Taylor and Schuster 2004), and Michigan (Creaser 1931). All adult males captured during this study were form I. Given that females were ovigerous in early spring and males enter the winter season as form I, mating in this species likely occurs in the fall or winter along the floodplain.

Fallicambarus fodiens females carrying instars were observed nocturnally foraging in open water on 18 March 2004 and 4 April 2009 in Greenbottom Wildlife Management Area. Several individuals (n = 8) were resting or grazing at their burrow entrances on periphyton that had colonized submerged canary grass (Phalaris canariensis L*.*). Within the colony burrows were flooded by 15–30 cm of standing water. Instars were observed leaving burrow entrances to graze. Upon provocation, disturbed females stopped moving long enough to allow instars to reattach to their abdomens, then retreated to their burrows. Seasonal data for Fallicambarus fodiens is presented in Table 3. Crayfish associates collected with Fallicambarus fodiens include Cambarus bartonii cavatus, Cambarus thomai, Orconectes virilis, and Procambarus acutus acutus.

#### Conservation status within study area.

Fallicambarus fodiens warrants conservation attention and is deserving of S1 status. Surveys are needed along the Kanawha River in wetland habitats to determine if this species persists there. Along the floodplain threats to this species’ survival include land use practices and their associated pollutants, hard surface run off, and destruction of bottomland forests.

### Orconectes (Crockerinus) obscurus

(Hagen, 1870)

Cambarus obscurus Hagen 1870:69, figs. 72–75, 154.
Cambarus propinquus  var. *obscurus*Faxon 1885:92.
Cambarus propinquus obscurus Hay 1899:960.
Cambarus (Faxonius) obscurus Ortmann 1905b:112; 1906:369, figs. 1, 2, 7.
Faxonius obscurus  Creaser 1933:5.
Faxonius (Faxonius) obscurus Creaser 1933a:5.
Orconectes obscurus Hobbs 1942a:352; 1974:36, fig. 117. Crocker 1957:36, 53, 75, figs. 5–6. Fitzpatrick 1963:61; 1967:160, figs. 3, 11–15, 25. Taylor et al. 1996:31. Taylor et al. 2007: 384.
Orconectes (Orconectes) obscurus Hobbs 1942b: 154.
Orconectes (Crockerinus) obscurus Fitzpatrick 1987:50. Hobbs 1989: 36, fig. 155. Jezerinac et al. 1995:26–34, figs. 11a–11h. Loughman 2010: 50–53, fig. 16.

#### Diagnosis.

Rostrum with slightly converging margins, not thickened, with marginal spines or tubercles; median carina absent; postorbital ridges possessing a sharp spine. Cephalothorax ovoid, slightly, dorsoventrally compressed, without setae. Areola 3.7–6.6 times longer than wide, comprising 27–39% of TCL, with 2–3 rows of punctations across narrowest region; cervical groove interrupted just above cervical spine; lacking hepatic spines; suborbital angle obsolete. Antennal scale about 1.5 times as long as wide; basiopodite spine of antenna well developed. Ischiopodite of antenna without spine. Chelae smooth, broad and robust, length 91% of TCL; mesial surface of palm consisting of two well developed rows of tubercles; mesialmost row consisting of 7–11; dorsolateral row with 5–11; lateral margin of propodus smooth, dorsal surfaces of both dactyl and fixed finger of propodus with weak dorsolateral ridges; some elongate setae at base of fixed finger. First form gonopods short, comprising 33% of TCL, with two terminal elements about equal length; corneous central projection comprising 23% of pleopod length, tapering distally to point; mesial process non-corneous, spatulate, partially surrounding central projection; cephalic base of central projection with right angle shoulder. Form two male gonopod non-corneous, blunt, shoulder not prominent or absent. Female annulus ventralis deeply embedded in sternum, moveable, wider than long, cephalolateral prominences well developed, distinctly separated by a trough; fossa rather deep, sinus sinuate in caudal 67% of annulus.

#### Color in life.

Carapace, abdomen and dorsal surface of chelae brown; rostral margins, caudal edge of carapace, and anterodorsal surface of terga dark brown; tips of chelae and knob at base of dactyl orange; tubercles on mesial and lateral margins of dactyl, mesial margin of palm, and mesial margin of propodus, and two spines or tubercles on anterodorsal surface of carpus yellow; reddish stripe on lateral margin of chelae; ventral margins beige.

#### Specimens examined.

Orconectes obscurus were collected from six counties at 16 locations in the current study, as listed below.

**BROOKE COUNTY:** Buffalo Creek at RT 2 crossing in Wellsburg, 40.261375 -80.61508; 4 September 2005 – (WLU 05090402), 2 I♂, 3 ♀. Cross Creek at entrance to Bruin Drive adjacent to Brooke High School, 40.306442 -80.5997; 28 June 2005 – (WLU 05062803), 1 ♀; 4 September 2005 – (WLU 05090403), 2 I♂, 1 II♂, 2 ♀. (3.) RT 2 crossing of nameless tributary 2.27 km (1.41 mi) S of Beech Bottom, 40.23163 -80.6523; 28 June 2005 – (WLU 05062801), 4 II♂. RT 2 crossing of nameless tributary in Beech Bottom proper, 40.306442 -80.5997; 28 June 2005 – (WLU 05062801), 1 ♀. **HANCOCK COUNTY:** Hardin Run 0.81 km (0.5 mi) from CR 2-7/RT 2 intersection on CR 2-7, 40.533314 -80.60326; 23 August 2005 – (WLU 05082302), 2 I♂, 1 ♀. Holbert Run 1.61 km (1.0 mi) from CR 2-8/ RT 2 intersection adjacent to CR 2-8, 40.474045 -80.58584; 23 August 2005 – (WLU 05082303), 1 I♂, 1 II♂, 1 ♀. Kings Creek at RT 2 crossing, 40.435715 N / 80.592514 W; 17 October 2005 – (WLU 05101701); 3 I♂, 4 ♀. Tomlinson Run backwater at RT 2 crossing, 40.54026 -80.628075; 8, March 2005 – (WLU 06030801), 9 I♂; 30 March 2005 – (WLU 06033001), 1 I♂. **MARSHALL COUNTY:** Big Grave Creek at Ohio River confluence in Moundsville, 39.9046 -80.75731; 20 July 2005 – (WLU 05072003 1 ♀; 4 September 2005 – (WLU 05090401), 2 I♂, 1 II♂. Fish Creek at RT 2 crossing, 39.808643 -80.81616; 30 October 2005 – (WLU 05103002), 7 I♂, 2 ♀; 30 April 2006 – (WLU 06043001), 8 O♀. Little Grave Creek at RT 2 crossing in Moundsville, 39.920944 -80.748566; 28 July 2007 – (WLU 05072807), 1 I♂, 1 II♂, 4 ♀. Nameless tributary at RT 2 crossing adjacent to Columbia Chemical operations, 39.85933 -80.79305; 28 July 2007 – (WLU 05072809), 2 I♂. **OHIO COUNTY:** Short Creek at RT 2 crossing, 40.18312 -80.676865; 4 September 2005 – (WLU 05090404), 2 I♂, 2 ♀. **PLEASANTS COUNTY:** Ben’s Run at RT 2 crossing, 39.46337 -81.08457; 28 July 2005 – (WLU 05072802), 1 I♂, 2♀. **WETZEL COUNTY:** Fishing Creek at RT 2 crossing, 39.63576 N/ -80.85848; 20 July 2005 – (WLU 05072001), 2 II♂, 1 ♀. Proctor Creek at RT 2 crossing, 39.70037 N/ -80.81791 W; 20 July 2005 – (WLU 05072001), 2 II♂, 3 ♀.

#### Distribution.

Orconectes obscurus occurs in north-west New York south through western Pennsylvania and north-central West Virginia, east to Maryland’s portion of the Youghiogheny River system and west to the Flushing Escarpment of Ohio (Hobbs 1989). Ontario populations are considered introduced (Taylor et al. 2007). Orconectes obscurus distribution in western West Virginia appeared to be limited to the Upper Ohio North and Upper Ohio South drainages (Jezerinac et al. 1995). Jezerinac et al. (1995) reported the southern extent of Orconectes obscurus range adjacent to the Ohio River as Proctor Creek at the Marshall/Wetzel counties line, and documented Orconectes sanbornii replacing Orconectes obscurus inFishing Creek, Wetzel County. Jezerinac et al. (1995) also documented Orconectes sanbornii as the dominant orconectid for the Middle Ohio North, Middle Ohio South, and Lower Ohio basins.

**Figure 19.**
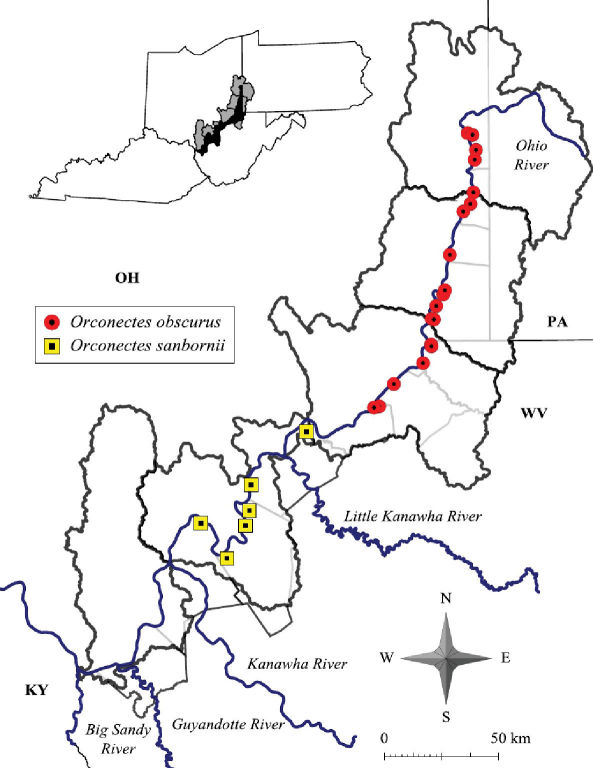
Orconectes obscurus and Orconectes sanbornii distribution along the West Virginia portion of the Ohio River floodplain

Orconectes obscurus has undergone a southern range expansion since Jezerinac’s surveys in the 1980’s (Figure 19). The southern extent of its range currently is Ben’s Run, Tyler County. Orconectes sanbornii’s northern limit currently is Middle Island Creek, due north of Saint Mary’s, Pleasant County. Orconectes obscurus and Orconectes sanbornii divide the Middle Ohio North basin, with Orconectes obscurus inhabiting northern portions of the basin and Orconectes sanbornii inhabiting southern portions (Figure 19).

The southward expansion of the range of Orconectes obscurus could be natural or an anthropogenic event. Orconectes are used as bait because of their ease of capture and high densities (Distefano et al. 2009b), so bait bucket release may explain the southward range expansion. Orconectes obscurus has a history as an invasive, with such populations present in New York and Ontario, Canada (Crocker and Barr 1968; Taylor et al. 1996). Many of the streams containing Orconectes obscurus populations in the southern region of the Middle Ohio North basin are second or third order streams that do not harbor large game fish populations.

Two alternative hypotheses explaining this expansion may include previous misidentification and natural expansion. Orconectes obscurus may have always been present historically where the species was collected in this survey, and misidentified by previous investigators. It is also possible that the species has expanded under natural conditions southward since the 1980’s, specifically invading the Hannibal Pool of the Ohio River and replacing Orconectes sanbornii in the mainstem. After displacement of Orconectes sanbornii in the Ohio River mainstem, additional streams could be colonized via stream confluences.

#### Morphometrics.

The largest individual was a 39.0 mm TCL form I male collected in Tomlinson Run backwater in Hancock County. The largest female was collected from the confluence of Little Grave Creek and the Ohio River in Marshall County and possessed a 37.4 mm TCL . Mean TCL for the species was 29.2 mm (n = 82, SE = 8.77). Sexual dimorphism is displayed in this species, with form I and form II male chelae significantly larger (t(345) = 6.8201, p = 0.0001) than female chelae. Morphometric data for Orconectes obscurus is presented in Table 8.

**Table 8.** West Virginia Ohio River floodplain Orconectes obscurus morphometrics.

Sex	N	Minimum	Maximum	Mean	Standard Deviation
Male I					
Carapace Length	35	8.4	39	29.85	6.7
Palm Length	35	3.5	9.01	7.57	1.89
Areola Width	35	1.0	2.63	1.88	0.61
Areola Length	35	6.1	13	10.14	2.81
Male II					
Carapace Length	12	9.2	30.8	24.7	7.0
Palm Length	12	3.5	7.5	4.9	1.3
Areola Width	12	1.2	2.1	1.5	0.3
Areola Length	12	4.4	10.1	7.0	1.5
Female					
Carapace Length	35	6.9	37.4	30.3	5.3
Palm Length	35	3.9	8.6	6.3	1.7
Areola Width	35	1.9	2.4	2.1	0.46
Areola Length	35	9.7	37.5	10.1	7.2

#### Habitat and natural history.

**Figure 20.**
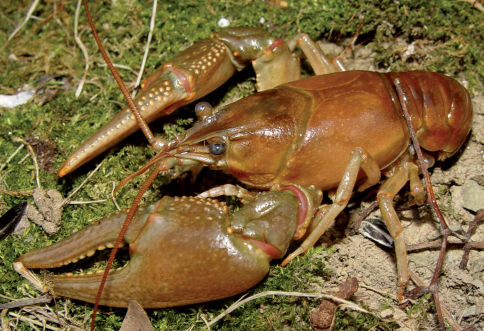
Orconectes obscurus, Upper Ohio North basin, Ohio County, West Virginia - WLU 05072807

Orconectes obscurus (Figure 20) occupy stream habitats throughout the central and northern regions of the floodplain. Habitats include first through fifth ordered streams and Ohio River backwaters. Healthy populations of Orconectes obscurus occur in all 3_rd_ through 5_th_ ordered streams from Ben’s Run, Tyler County, north to Tomlinson Run, Hancock County. Orconectes obscurus were frequently collected from streams within two specific macrohabitats. Slab boulders and leaf packs were utilized by all demographics; form I males were associated primarily with slab boulders. Leaf packs in pool thalwegs were utilized with increased frequency by Orconectes obscurus juveniles. Based on captive observations, leaf packs offer both structural protection and periphyton for foraging (Z. Loughman personal obs.).

Orconectes obscurus is also a tertiary burrower, creating minimal burrows under substrate items. Small gravel and cobble piles usually were present along margins of slab boulders harboring Orconectes obscurus. One significant behavioral difference between the genera Orconectes and Cambarus along the floodplain is the difference in expressed territoriality. Orconectes obscurus and other orconectids displayed limited territorality. In one instance in Cross Creek, Brooke County, 11 individuals were collected from a single slab boulder.

Orconectes obscurus were collected from two ephemeral streams. This habitat has not previously been reported for the species, and is rarely reported forany Orconectes species(Distefano et al. 2009a ). In Brooke County, Orconectes obscurus were observed in a first order stream tributary to the Ohio River mainstem, foraging on large mats of Cladophora spp. In another headwater stream in Brooke County, they were collected 1.5 km from the river mainstem and had traversed three 1.0 m waterfalls and their associated plunge pools. It is likely that these crayfish inhabit the mainstem of the river and had migrated into the stream, returning to the river during periods of drawdown.

Jezerinac et al. (1995) described Orconectes obscurus’s and Orconectes sanbornii’s life cycle in West Virginia. Form I males are present from fall into winter and mate in the early spring. After spring mating, males molt into second form in late June and proceed throughout most of the summer in this condition (Table 9). Life history data collected during this study validate Jezerinac et al.’s (1995) findings. Beginning in late April and continuing through mid May, females extruded eggs and carried instars. Ovigerous females were collected on 8, 9, and 12 May 2007. Pleopodal egg counts averaged 113 (n = 8 females, SE = 18.2) with a mean egg diameter of 1.7 mm. There was no correlation between pleopodal egg number and TCL (r2 = 0.88, n = 8). Beginning in late June of both 2005 and 2006, young of the year were frequently captured, indicating their release from female’s pleopods. Males in late July and early August underwent a late summer molt into first form condition (Table 9). After this molt, mating effort increased through late summer and fall until winter hibernation. In addition to the late summer molt, males molted in mass during May at the same time that females became ovigerous. This life history mirrors that of Ohio populations as well (Fielder 1972). Crayfish associates collected with Orconectes obscurus include Cambarus carinirostris, Cambarus bartonii cavatus, Cambarus robustus, and Cambarus thomai.

**Table 9.** West Virginia Ohio River floodplain Orconectes and Procambarus seasonal demographics.

Species	J	F	M	A	M	Jn	J	A	S	O	N	D
Orconectes (Cambarus) obscurus												
Male 1			×				×	×	×			
Male 2						×	×		×			
Females						×	×	×	×			
Ovigerous Females				×	×							
Orconectes (Cambarus) sanbornii												
Male 1		×	×				×					
Male 2					×		×					
Females							×					
Ovigerous Females												
Orconectes (Gremicambarus) virilis												
Male 1				×	×		×			×		
Male 2				×	×				×	×		
Females					×		×			×		
Ovigerous Females												
Orconectes (Procambarus) rusticus												
Male 1		×	×									
Male 2												
Females		×	×				×					
Ovigerous Females		×	×									
Procambarus (Orconectes) acutus												
Male 1		×	×	×	×							
Male 2		×	×	×								
Females			×	×	×							
Ovigerous Females			×									

#### Conservation status within study area.

Orconectes obscurus populations are stable and expanding southward along the floodplain. Determining if this expansion is a natural or anthropogenic event is important for conservation of any crayfish species that Orconectes obscurus may ultimately extirpate.

### Orconectes (Crockerinus) sanbornii

(Faxon, 1884)

Cambarus Sanbornii Faxon 1884:128.
Cambarus propinquus sanbornii Faxon 1885:91, figs. 3, 10.
Cambarus propinquus var. *sanbornii*Underwood 1886:372.
Cambarus propinquus  var. *sanbornii*Osborn and Williamson 1898:21
Cambarus propinquus sanbornii Faxon 1898:660.
Cambarus propinquus sanborni Ortmann 1905b:128.
Cambarus (Faxonius) propinquus sanbornii Ortmann 1906:365.
Cambarus obscurus sanbornii Ortmann 1906:437.
Faxonius sanbornii Creaser 1933a:3.
Faxonius (Faxonius) sanbornii Creaser 1933b:21.
Orconectes propinquus sanbornii Hobbs 1942a: 352. Fitzpatrick 1963:61.
Orconectes (Orconectes) propinquus sanbornii Hobbs 1942b :154.
Faxonius sanborni sanborni  Creaser 1962:2.
Orconectes sanbornii sanbornii Fitzpatrick 1967:157, figs. 2–18, 23.
Orconectes sanbornii  Stevenson 1967:208. Taylor et al. 1996:31. Taylor et al. 2007:385.
Orconectes sanbornii sanbornii Hobbs 1974: 40, fig. 121.
Orconectes (Crockerinus) sanbornii sanbornii Fitzpatrick 1987:50, fig 3. Hobbs 1989:37, fig.159.
Orconectes (Crockerinus) sanbornii Jezerinac et al. 1995:35–43, figs. 15a–15h. Taylor and Schuster 2004:196–198, figs. 166, 167A–167G.

#### Diagnosis.

Rostrum with slghtly converging margins, not thickened, with marginal spines or tubercles; median carina absent. Cephalothorax ovoid, slightly, dorsoventrally compressed, without setae. Areola 3.4–9.3 times longer than wide, comprising 31–37% of TCL, with 2–3 rows of punctations across narrowest region; cervical groove interrupted just above cervical spine; lacking hepatic spines; suborbital angle obsolete. Antennal scale about 1.5 times as long as wide; basiopodite spine of antenna well developed; ischiopodite of antenna without spine. Chelae smooth, broad and robust, length 85% of TCL; mesial surface of palm with two well developed rows of tubercles; mesialmost row consisting of 7–11 tubercles; dorsolateral row with 7–11; lateral margin of propodus smooth, dorsal surfaces of both dactyl and fixed finger of propodus with weak dorsolateral ridges; some elongate setae at base of fixed finger. First form male gonopods short, comprising 30% of TCL, with two terminal elements about subequal length; corneous central projection comprising 16% of pleopod length, tapering distally to point; mesial process non-corneous, spatulate, partially surrounding central projection; cephalic base of central projection sloping, without right angle shoulder. Form two male gonopod non-corneous, blunt, shoulder not prominent or absent. Female annulus ventralis deeply embedded in sternum, moveable, wider than long, cephalolateral prominences flattened; fossa and sulcus shallow; sinus straight.

#### Color in life.

Carapace, abdomen and dorsal surface of chelae brown; rostral margins, caudal edge of carapace, and central surface of terga dark brown; tips of chelae and tubercles at dactyl base orange; tubercles on mesial and lateral margins of dactyl, mesial margin of palm, and mesial margin of fixed finger of propodus, and two spines or tubercles on anterodorsal surface of carpus, yellow; reddish stripe on lateral margin of chelae; ventral margins beige.

#### Specimens examined.

Orconectes sanbornii were collected from four counties at seven locations in the current study, as listed below.

**JACKSON COUNTY:** Little Mill Creek at crossing of RT 33 N 9.43 air km (5.86 mi) N of Ravenswood, 38.86171 -81.85407; 20 July 2006 – (WLU 06072001), 1 II♂, 2 ♀. Little Sandy Creek at intersection of RT 68/CR 8, 38.991497 -81.761765; 21 July 2006 – (WLU 06072103), 1 I♂, 1 II♂, 11 ♀, 4 Juv. **MASON COUNTY:** Sliding Creek at intersection of CR 4/RT 33, 38.999382 -81.987686; 20 July 2006 – (WLU 06072002), 2 I♂, 1 ♀. **PLEASANTS COUNTY:** Middle Island Creek at RT 2 crossing, 39.40328 -81.197624; 21 July 2006 – (WLU 06072104), 1 II♂, 2 ♀. **WOOD COUNTY:** Big Run at CR 21-1 crossing, 39.364048 -81.45656; 21 July 2006 – (WLU 06072106), 3 II♂, 5 ♀, 1 Juv. Lee Creek at CR 11 crossing, 39.153275 -81.73507; 21 July 2006 – (WLU 06072102), 1 II♂, 2 ♀. Nameless tributary crossing 3.54 km (2.2 mi) S of Parkersburg, 39.05142 -81.742836; 21 July 2006 – (WLU 05072105), 1 II♂, 1 ♀.

#### Distribution.

Orconectes sanbornii occurs throughout the Middle Ohio river drainage in Ohio, West Virginia and Kentucky (Taylor and Schuster 2004). Sites harboring Orconectes sanbornii are shown in Figure 19. The most northern historic populations of the species on the floodplain occurred in Fishing Creek, Wetzel County were recently replaced by southward expanding Orconectes obscurus populations. Orconectes sanbornii is absent from lower reaches of Fishing Creek associated with the confluence of the Ohio River, and still is present in mid- and headwater sections of Fishing Creek (Loughman, unpublished data). Currently, Orconectes sanbornii inhabits the Middle Ohio North, Middle Ohio South, and Lower Ohio basins (Figure 19). Within these basins, Orconectes sanbornii is the only native orconectid.

#### Morphometrics.

The largest individual was a female with a 35.5 mm TCL from West Creek, Jackson County. The largest male was a form I with a TCL of 34.3 from West Creek. The mean TCL for Orconectes sanbornii was 24.15 mm (n = 35, SE = 8.91). Morphometric data for Orconectes sanbornii is presented in Table 9.

#### Habitat and natural history.

**Figure 21.**
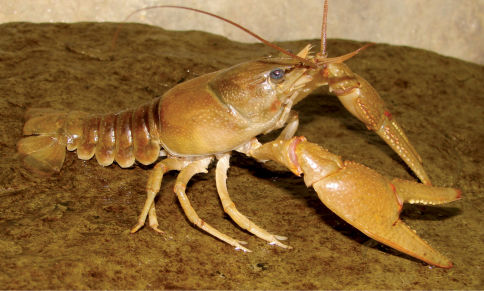
Orconectes sanbornii, Middle Ohio South, Jackson County, West Virginia – WLU 06072103

Orconectes sanbornii (Figure 21) habitat requirements were similar to those of Orconectes obscurus. Orconectes sanbornii is typical of tertiary burrowing crayfish, and inhabited small-to large-sized streams. Slab boulders, leaf packs, and depositional environments were all habitats used by the species. Based on this study, the life cycle of Orconectes sanbornii mirrors that of Orconectes obscurus (Table 9).

**Table 10.** West Virginia Ohio River floodplain Orconectes sanbornii morphometrics.

Sex	N	Minimum	Maximum	Mean	Standard Deviation
Male I					
Carapace Length	3	24.6	34.3	27.9	2.4
Palm Length	3	5.6	10.2	7.4	1.1
Areola Width	3	1.4	2.1	1.6	0.1
Areola Length	3	8.4	2.3	9.8	1.0
Male II					
Carapace Length	8	11.5	34.0	20.9	7.9
Palm Length	8	2.0	3.0	3.8	1.9
Areola Width	8	1.0	1.6	1.2	0.4
Areola Length	8	4.0	11.8	7.1	3.0
Female					
Carapace Length	24	12.2	35.5	25.6	5.7
Palm Length	24	1.0	7.2	4.5	1.4
Areola Width	24	0.5	6.0	1.7	0.8
Areola Length	24	3.8	12.6	8.6	2.1

Orconectes sanbornii demonstrated the same gregarious behavior observed in Orconectes obscurus. Behavioral differences observed between Orconectes obscurus and Orconectes sanbornii specifically differed in the use of stream cover.In West Creek, Jackson County, Orconectes sanbornii were observed exposed in the stream channel. Orconectes sanbornii used interstitial spaces between boulder margins, and the majority of individuals were collected from exposed areas. Orconectes sanbornii were exposed mid-channel resting on the stream bottom with their antennae held posteriorly over their cephalothorax, and would not seek cover until prodded. Orconectes were noted for using cover less than cambarids in this study, but the extreme level of behavior observed in Orconectes sanbornii warrants special mention. Crayfish associates collected with Orconectes sanbornii include Cambarus bartonii cavatus and Cambarus thomai.

#### Conservation status within study area.

The ramifications of Orconectes obscurus expansion southward into historic ranges of Orconectes sanbornii remains to be seen. This interaction needs to be monitored to determine the true relationship between these sibling species.

### Orconectes (Gremicambarus) virilis

(Hagen, 1870)

Cambarus virilis Hagen 1870:63, fig. 23–28.
Cambarus wisonsinensis Bundy 1876:4.
Cambarus debilis Bundy 1876:24.
Cambarus cousii Streets 1877:803.
Cambarus Cousei Faxon 1885:97.
Cambarus wisconsiensis Harris 1900:271.
Cambarus cousei Harris 1903:134.
Cambarus viridis Moenkhaus 1904:111.
Cambarus (Faxonius) virilis Ortmann 1905b:113. Creaser 1932:326, fig. 1, 2, 8.
Faxonius virilis Creaser 1933a:3; 1962:2.
Faxonius (Faxonius) virilis Creaser 1933b:21
Orconectes virilis Hobbs 1942a:352; 1972:91, figs. 72h, 73e; 1974:42, fig. 162. Fitzpatrick 1963:61. Page 1985:417, fig. 151–154. Pflieger 1987:22. Pflieger 1996:122–126, fig. 28A–28I. Taylor et al. 1996:31. Taylor et al. 2007.
Orconectes (Orconectes) virilis Hobbs 1942b:154.
Orconectes (Gremicambarus) virilis Fitzpatrick 1987:54, fig. 5. Hobbs and Jass 1988:79–86, figs. 52a–52o. Hobbs 1989:42, fig. 199. Jezerinac et al. 1995:44–51, figs. 19a–19h. Loughman 2010:53–57, fig. 18.

#### Diagnosis.

Rostrum with straight margins, not thickened or possessing spines or tubercles; median carina absent; postorbital ridges terminating cephalically with spine or tubercle. Branchiostegal spine reduced; hepatic spine absent. Cephalothorax oval shaped and slightly dorsoventrally flattened in profile; without setae; suborbital angle obsolete. Areola 7.1–19.0 times longer than wide, comprising 34–39% of TCL, with 1–2 rows of punctations across narrowest region. Chelae smooth, broad and robust; mesial surface of palm with two rows of defined tubercles; first row with 6–8 rounded tubercles; second with 5–8 tubercles; lateral margin of propodus smooth; dorsal surfaces of both dactyl and fixed finger of propodus with prominent well developed longitudinal ridges; elongate plumose setae at base of fixed finger of propodus. First form male gonopods long, comprising 42% of TCL, with 2 terminal elements, both bent and curving at about 30° to the base; central projection corneous, comprising 24% of gonopod length, cephalic base without shoulder. Form two male gonopod noncorneus, gently curving caudally; mesial process subequal in length to central projection, blunt. Female annulus ventralis rhomboid, fossa large, sulcus wide, cephalolateral prominences weak, sinus only evident on caudal surface.

#### Color in life.

Carapace and abdomen dorsally olivaceus or brown; rostral margins darker brown to black; postorbital ridges and caudal margins of cephalic portion of carapace along cervical groove brown; two rows of blotches on dorsal surface of abdomen; dorsal surface of chelae emerald green; tips of propodus and dactyl darker green; all knobs on chelipeds beige or tan; ventral surfaces cream or white.

#### Specimens examined.

Orconectes virilis were collected in Mason and Pleasants counties at three locations in the current study, as listed below.

**MASON COUNTY:** Krodel Park marsh adjacent to Fort Randolph reproduction, 38.785404 -82.12209; 26 March 2004 – (WLU 04032601), 2 I♂, 1 ♀; 28 April 2004 – (WLU 04042801), 1 II♂, 1 ♀; 17 March 2005 – (WLU 05031703), 1 I♂; 28 March 2005 – (WLU 05032802), 7 I♂; 5 May 2005 – (WLU 05050501), 5 II♂, 1 ♀. Pin oak swamp adjacent to Point Pleasant Moose Lodge in Wagner, 38.833603 -82.12227; 27 February 2005 – (WLU 05022701), 1 I♂. **PLEASANTS COUNTY:** Ohio River embayment 4.03 air km (2.52 mi) S of St. Mary’s, 39.397575 -81.202415; 30 March 2004 – (WLU 04033002), 2 II♂.

#### Distribution.

Orconectes virilis native range includes Saskatchewan south through Montana and Utah east to Ontario, and throughout northern portions of the Mississippi River system. Several disjunct populations persist in Ohio and throughout the northeast.West Virginia Orconectes virilis populations are invasive (Jezerinac et al. 1995). Orconectes virilis were limited to two sites in the Middle Ohio North and Middle Ohio South basins (Figure 10). The Middle Ohio South population is present in an Ohio River embayment at the northern city limit of Saint Mary’s, Pleasant County. The Lower Kanawha basin population occurs in Krodel Park lake, Point Pleasant, Mason County. The Saint Mary’s population does not appear to be abundant, with three individuals collected during seven collection events in 2004 and 2005. The Krodel Park population represents a potential source population for future invasions.

#### Morphometrics.

The largest observed individual was a 52.4 mm TCL form I male collected from Krodel Park Lake, Mason County. The largest female was 43.3 TCL, also from Krodel Park. The mean TCL for Orconectes virilis was 42.8 mm (n = 22, SE = 6.11). This species was the largest crayfish collected in this study. Morphometrics for Orconectes virilis are presented in Table 11.

**Table 11.** West Virginia Ohio River floodplain Orconectes virilis morphometrics.

Sex	N	Minimum	Maximum	Mean	Standard Deviation
Male I					
Carapace length	11	38.5	52.4	44.93	3.96
Palm Length	11	7.2	12.67	10.23	1.57
Areola Width	11	1.1	2.55	1.57	0.37
Areola Length	11	7.2	11.58	8.86	1.32
Male II					
Carapace length	8	28.3	51.0	41.3	2.3
Palm Length	8	7.9	34.6	15.6	9.6
Areola Width	8	1.6	2.5	2.0	0.3
Areola Length	8	5.0	10.0	7.9	2.1
Female					
Carapace length	3	35.5	43.3	39.3	5.6
Palm Length	3	8.9	31.8	20.4	16.1
Areola Width	3	1.4	1.8	1.67	0.3
Areola Length	3	2.1	7.2	7.2	0.1

#### Habitat and natural history.

**Figure 22.**
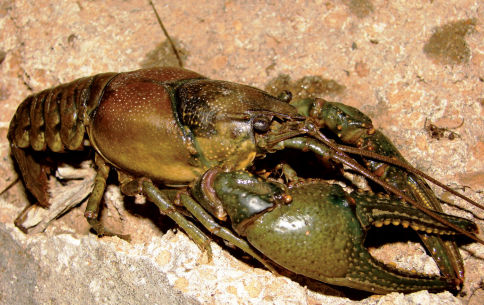
Orconectes virilis, Middle Ohio South basin, Mason County, West Virginia – WLU 04032601

Orconectes virilis (Figure 22) is an invasive floodplain species; the closest native populations are endemic to the upper Mississippi River valley (Hobbs and Jass 1988; Page 1985). Two disjunct populations were discovered along the Ohio River floodplain, in Krodel Park, Mason County and near Saint Mary’s, Pleasant County, in an Ohio River embayment. Krodel Lake population stock undoubtedly came from bait-bucket introductions. Less than five km from Krodel Lake is an aquaculture facility that raises and sells Orconectes virilis for fish bait. Discussions with anglers informed the primary author that “soft craws” were purchased from local bait dealers and used in Krodel Park. Orconectes virilis has been collected from six wetlands surrounding Krodel Lake. All of these sites are within one km of the lake proper. Within the lake, Orconectes virilis uses riprap in the littoral zone for cover. Over one hundred adults were observed utilizing this habitat between 20:00–23:00 h on 5 May 2005.

In certain situations Orconectes virilis travels as far as one km from the lake to nearby wetlands. Its presence in a vernal pool system with zero fishing effort shows the propensity of this species to migrate. In one instance, Orconectes virilis had not been captured from an ephemeral pool system in spring and summer of 2004. After severe flooding in the fall of 2004, in which Krodel Lake spilled over into nearby bottomland forest, Orconectes virilis was captured in these wetlands. Life history information for invasive populations in West Virginia is unknown. Available life history information is presented in Table 9. Orconectes virilis are very large crayfish, and it is not hard to understand why they are capable of displacing native West Virginia species. The chelae on form I males in many instances were longer than the total body length of native Orconectes sanbornii.

#### Conservation status within study area.

Orconectes virilis populations require monitoring. This invasive species has proven to be successful in destroying several mid-Atlantic crayfish populations (Jezerinac et al. 1995, Kilian et al. 2010, Loughman and Welsh 2010, Swecker et al. 2010). The Krodel Lake population represents an important potential source population for future introductions.

### Orconectes (Procericambarus) rusticus

(Girard, 1852)

Cambarus rusticus  Girard 1852:88. Faxon 1885:108, fig. 8.
Cambarus juvenilis Hagen 1870:66, figs. 29–33, 157.
Cambarus (Faxonius) rusticus Ortmann 1905b:112.
Faxonius rusticus Williamson 1907:753. Creaser 1933a:5.
Cambarus (Faxonius) rusticus rusticus Ortmann 1931:82.
Cambarus (Faxonius) juvenilis Ortmann 1931:84 [in part].
Faxonius (Faxonius) rusticus rusticus Creaser 1933b:21.
Orconectes rusticus rusticus Hobbs 1942a:352. Fitzpatrick 1963:61.
Orconectes (Orconectes) juvenilis Hobbs 1942b:154.
Orconectes rusticus Pennak 1953:465. Hobbs 1972:92, figs. 74c, 75b–d. Page 1985:412, figs 145–147. Taylor et al. 1996:31. Taylor et al. 2007:385.
Procambarus rusticus  Huner 1978:4.
Orconectes (Procericambarus) rusticus Fitzpatrick 1987:58. Hobbs and Jass 1988:66–78, figs. 46a–46m, 47–49. Hobbs 1989:49, fig. 174. Jezerinac et al. 1995:52–58, figs 23a–23h. Taylor 2000:132. Taylor and Shuster 2004:192–195, figs. 163, 164A–164G.

#### Diagnosis.

Rostrum with concave margins, not thickened, with spines or tubercles; median carina absent; mandible with smooth cutting edge. Cephalothorax oval, slightly dorsoventrally compressed in profile. Areola 4.6–19.4 times longer than wide, comprising 36–39% of TCL, with 2–3 rows of punctations across narrowest region; branchiostegal spine poorly developed; suborbital angle obsolete or poorly developed. Chelae robust; mesial surface of palm with two rows of defined tubercles, first row with 5–9 tubercles; second row with 4–9 tubercles of smaller diameter. First form male gonopods long, comprising 26% of TCL, with 2 straight terminal elements; central projection comprising 56% of gonopod length; well developed shoulder at cephalic base of central projection. Second form male gonopod non-corneous, straight, mesial process slightly subequal in length to central projection, blunt, shoulder not evident. Annulus ventralis rhomboid in shape, fossa moderately large, cephlolateral prominences well developed, trough narrow, sinus evident on caudal surface.

#### Color in life.

Carapace and abdomen dark green or brown; large rusty lateral blotch present on each side of posterior margin of carapace; fixed finger of propodus and dactyl with red tips and black band; ventral surfaces cream or white.

#### Specimens examined.

Orconectes rusticus were only collected from Marshall County at two locations in the current study, as listed below.

**MARSHALL COUNTY:** PPG Wildlife Management Area adjacent to RT 2 S, 39.736244 -80.84638; 21 March 2006 – (WLU 06032103), 1 I♂; 18 April 2006 – (WLU 06041802) 4 I♂. **WETZEL COUNTY:** Ohio River backwater at Marshall/Wetzel County line, 39.717846 -80.514959; 2 April 2004 – (WLU 04040203), 4 I♂, 3 O♀; 11 April 2004 – (WLU 04041101), 3 I♂, 3 O♀; 21 March 2006 – (WLU 06032101), 10 I♂, 1 O♀; 18 April 2006 – (WLU 06041801) 9 I♂, 3 ♀.

#### Distribution.

**Figure 23.**
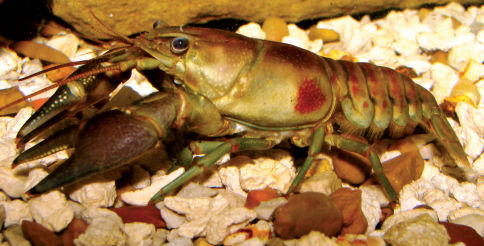
Orconectes rusticus, Middle Ohio North basin, Marshall County, West Virginia – WLU 04040203

Orconectes rusticus (Figure 23)is native to lower and central portions of the Ohio River system in Kentucky, Ohio, and Indiana north to western portions of Lake Erie in southeastern Michigan and north western Ohio (Taylor 2000.), and is one of two invasive crayfish species in West Virginia. Prior to this survey, it appeared to be limited to Four Pole Creek in Huntington, and isolated sections of the Kanawha and Little Kanawha River systems. Both floodplain populations are allied with the upper Ohio River South basin in the northern panhandle (Figure 10) and are associated with Ohio River embayments adjacent to industrial sites. Additional investigators discovered Orconectes rusticus populations throughout the Kanawha River system in recent years (Casey Swecker, Marshall University, personal communication).

The Upper Ohio South basin is the only basin within the floodplain that currently harbors Orconectes rusticus populations. Bayer Chemical Plant and Pittsburgh Paint and Glass (PPG) Chemical Plant both possess embayments connected to the Ohio River mainstem that contain Orconectes rusticus populations. The PPG population is present in a “pond” with a connection to the Ohio River mainstem. The Bayer population is present in a series of backwaters with mainstem connections. Trapping results show that the Bayer population has higher densities than the PPG population.

Why Orconectes rusticus is limited to these two backwaters despite its presence in the Ohio River mainstem needs furthur investigation. Populations present in the mainstem could operate as sources for future invasions into new watersheds. The extent of the range of Orconectes rusticus within the mainstem is also in need of future work. Given the Ohio River’s manipulation into a series of non-contiguous pools, investigations into those pools that harbor Orconectes rusticus populations and those pools that do not is a proactive move to understand basins at risk of future invasions.

#### Morphometrics.

Forty-four Orconectes rusticus were collected from two sites. The largest individual was an ovigerous female 44.1 mm TCL. The largest male was a 38.4 mm TCL form I male from PPG Wildlife Management Lake. Mean Orconectes rusticus TCL was 31.0 mm (n = 41, SE = 6.12). Nine females were ovigerous and had a mean carapace length of 30.1 mm. Morphometrics data for Orconectes rusticus is presented in Table 12.

**Table 12.** West Virginia Ohio River floodplain Orconectes rusticus morphometrics.

Sex	N	Minimum	Maximum	Mean	Standard Deviation
Male I					
Carapace Length	31	19.3	38.4	31.5	4.1
Palm Length	31	5.8	13.9	10.4	6.2
Areola Width	31	1.1	2.4	1.7	0.4
Areola Length	31	5.0	13.4	10.4	2.5
Male II					
Carapace Length	---	---	---	---	---
Palm Length	---	---	---	---	---
Areola Width	---	---	---	---	---
Areola Length	---	---	---	---	---
Female					
Carapace Length	10	18.3	44.01	29.0	7.3
Palm Length	10	2.0	3.0	8.9	6.1
Areola Width	10	1.1	3.5	4.0	6.5
Areola Length	10	3.2	15.4	8.6	3.5

#### Habitat and natural history.

In West Virginia’s Ohio River floodplain, Orconectes rusticus inhabits two Ohio River back- waters (Figure 10). Both embayments are nutrient rich, shallow, lentic systems with an abundance of detritus and algae. Nutrient-rich environments are preferred habitats of Orconectes rusticus and Ohio River backwaters provide ideal conditions for the species (Hobbs et al. 1989; Lodge et al. 2000a). All Orconectes rusticus collected in this study were trapped in late winter and early spring. Very little natural behavior was observed.

Life history parameters of the Bayer population were determined from specimens and a review of the literature. All males captured in traps in March and April were form I and possessed heavily encrusted carapaces. The level of encrustation is directly proportional to the length of time between molts (Hobbs 1981). Given the conditions of collected males, which in many instances were black and encrusted, individuals likely molted into form I the previous fall. Other Orconectes rusticus populations undergo a late summer/fall mating season, and it is likely that this population may mate during the fall as well (Jezerinac et al. 1995, Taylor and Schuster 2004).

Ovigerous females were collected on 2, 14, and 18 April 2006 (Table 9). Seventy percent of females were ovigerous at this time. Egg counts increased with female size and ranged from 75 ova for 26.5 mm TCL to 356 for a 35.9 mm TCL female. Mean egg diameter was 1.8 mm. There was not a correlation between egg number and TCL (r2 = 0.18, n = 7); however, this could possibly be an artifact from small sample size. Date of egg extrusion and counts are similar to native Kentucky populations at similar latitudes (Prins 1968). Females likely mate in the fall, hold active sperm inside spermatheca throughout the winter, and extrude eggs in late-March. Orconectes rusticus in previous life history studies were noted to undergo the typical Orconectes life history cycle, which has been explained in the Orconectes obscurus natural history section (Prins 1968; Capelli 1982; Jezerinac 1982).

Orconectes rusticus expansion into new territory was observed at the Bayer site. A headwater stream that was not connected to the Bayer series of backwaters became connected to this system in the spring of 2004. At this time extensive survey efforts were undertaken to determine if Orconectes rusticus was present within the stream; none were found. Ten months after initial surveys of this stream, Orconectes rusticus had migrated 2 km upstream.

#### Conservation status within study area.

Given the aggressive natureof this invasive species, annual monitoring efforts are warranted. The impact of this species on native crayfish communities in northern West Virginia is unknown and should be determined as soon as possible.

### Procambarus (Ortmannicus) acutus

(Girard, 1852)

Cambarus acutus  Girard 1852:91.
Astacus Blandigii  Leconte 1956:400.
Cambarus acutus  var. A Hagen 1870:36.
Cambarus acutus  var. B Hagen 1870:36.
Cambarus stygius Bundy 1876:3.
Cambarus Stygius Underwood 1886:373.
Cambarus blandingii acutus Faxon 1890:619.
Cambarus blandingi acutus Ortmann 1905a:105.
Cambarus (Cambarus) blandingi acutus Ortmann 1905a:105.
Ortmannicus blandingi acutus Rhoades 1942:1.
Procambarus (Ortmannicus) acutus acutus Hobbs 1972: 9; 1974:53, fig. 240; 1981:372, figs. 15b, 136a, 138b, 140, 143–145, 244. Hobbs and Jass 1988:105–113, figs. 69a–69j, 70.
Procambarus acutus Pflieger 1996:130–133, figs. 30A–30J. Taylor et al. 1996:31. Taylor et al. 2007:385.
Procambarus (Ortmannicus) acutus Page 1985:376, figs. 107A–107F. Taylor and Schuster 2004:203–205, figs. 172A–172B, 173A–173H. Loughman 2007:495; 2010:57, fig. 20.

#### Diagnosis.

Rostrum slightly broad and triangular; width of rostrum margins reduced; margins converging terminating in 2 marginal spines; acumen with distal rostral spine; postorbital ridges prominent, cephalic margin with tubercle; cephalothorax dorsolaterally compressed in profile, anterior portion vaulted; areola obliterated at narrowest point; branchiostegal region moderately punctate, with small tubercles; small cervical spine present; chelae elongate and lance shaped; mesial surface of palm with single dorsal row of 7–9 pronounced tubercles; additional tubercles scattered over dorsal surface of palm. Bases of first form male gonopods contiguous; gonopod with 4 terminal elements, covered by dense setae; central projection pointed and corneous; caudal process short; mesial process pointed and straight in profile; cephalic process elongated and pointed; second form gonopod annulus ventralis circular in shape, embedded deeply in sternum, and movable.

#### Color in life.

Carapace, chelae, and pereiopods dorsally and laterally red-gray, gray-purple, burgundy, or red; branchial region of smaller individuals mottled with black spots; tubercles on chelae cream, red-brown, red, or black; dorsal surface of abdomen with a distinct black wedge.

#### Specimens examined.

Procambarus acutus were collected in Mason County at three locations, listed below.

**MASON COUNTY:** Roadside ditch adjacent to RT 2, 9.17 km (5.7 mi) S of Point Pleasant, 38.80469 -82.18821; 12 April 2004 – (WLU 04041206), 1 I♂, 3 ♀; 13 April 2004 – (WLU 04041301), 1 II♂; 28 April 2004 – (WLU 04042802), 2 I♂, 1 II♂; 17 March 2005 – (WLU 05031701), 2 I♂; 18 March 2005 – (WLU 05031802), 2 II♂, 2 ♀. Krodel Park marsh adjacent to Fort Randolph reproduction, 38.785404 -82.12209; 5 March 2005 – (WLU 05030502), 1 I♂, 2 ♀; 17 March 2005 – (WLU 05031703), 8 I♂; 22 March 2005 – (WLU 05032201), 2 I♂, 2 II♂, 2 O♀, 8 ♀, 1 Juv. Pin oak swamp adjacent to Point Pleasant Moose Lodge in Wagner, 38.833603 -82.12227; 26 February 2004 – (WLU 04022601), 4 I♂, 2 ♀; 26 March 2004 – (WLU 04032601), 3 ♀, 2 Juv.; 12 April 2004 – (WLU 04041205), 4 I♂, 2 ♀, 2 Juv.; 28 April 2004 – (WLU 04042801), 9 II♂, 2 ♀; 21 March 2005 – (WLU 05032101), 1 ♀; 10 April 2005 – (WLU 05041001), 1 I♂, 1♀.

#### Distribution.

Procambarus acutus is a wide-ranging species associated with wetlands present throughout the central and eastern United State excluding the majority of the Appalachian Mountains (Taylor and Schuster 2004). Several introduced populations occur through North America (Taylor et al. 2007). In West Virginia, Procambarus acutus occurs in the Middle Ohio South and Lower Kanawha basins (Figure 16), and was first reported occurring in West Virginia by Loughman (2007). Given its history of introductions elsewhere, Procambarus acutus was initially thought to be an introduced species in West Virginia. It’s use in aquaculture and as bait for fishing has led to non-indigenous populations occurring throughout North America, with confirmed non-indigenous populations documented in California (Gander 1927), Maine (Crocker 1979), and Kentucky (Taylor and Schuster 2004). As discussed below, current evidence suggests that Procambarus acutus is a native species in West Virginia.

Several species, including the oak, Quercus bicolor Willd., the salamander, Ambystoma texanum (Mathes, 1885), and Fallicambarus fodiens, are found in the Lower Ohio and Lower Kanawha drainages in West Virginia. They are theorized to be pre-glacial Marietta River relicts (Green and Pauley 1987; Jezerinac and Stocker 1987; Strausbaugh and Core 1978). The Marietta River was a major tributary of the pre-glacial Teays River, and the area of the Ohio River and Kanawha River confluence is considered to be the Marietta River Valley (Stout et al. 1943). Several species that are found in no other part of the state inhabit this biotic region. Jezerinac and Stocker (1987) used the Marietta River Valley to explain the disjunction of the West Virginia Fallicambarus fodiens population from the core of its range across the midwest. This species occurs sympatrically with Procambarus acutus at two of three sites along the floodplain.

Ambystoma texanum (Green and Pauley 1987) occurred at two sites harboring Procambarus acutus populations. Both Ambystoma texanum and Fallicambarus fodiens have limited, disjunct ranges in West Virginia. In Ohio, Procambarus acutus has been collected from sites in the pre-glacial Teays River drainage, as well as in the area theorized to be the Teays River, Marietta River confluence (Thoma and Jezerinac 2000). The presence of these two species at three of the four Procambarus acutus collection sites, along with Procambarus acutus only being collected in the Marietta River Valley and nowhere else along the Ohio River floodplain of West Virginia, appears to validate the hypothesis that Procambarus acutus is native to West Virginia

#### Morphometrics.

The largest Procambarus acutus collected in this study was a 43.1 mm TCL female collected from the Moose Lodge wetland, Mason County. The largest male was a 40.6 mm TCL form I also collected from the same locality. Mean Procambarus acutus carapace length was 28.7 mm (n = 68, SD = 7.79). Morphometrics data for Procambarus acutus are presented in Table 13.

**Table 13.** West Virginia Ohio River floodplain Procambarus acutus morphometrics.

Sex	N	Minimum	Maximum	Mean	Standard Deviation
Male I					
Carapace Length	24	16.4	40.6	26.8	8.7
Palm Length	24	2.5	21.2	6.2	3.7
Areola Width	24	0.1	2.8	1.4	0.6
Areola Length	24	3.2	10.6	6.3	2.2
Male II					
Carapace Length	5	13.6	38.8	29.8	8.2
Palm Length	5	2.0	34.5	17.4	11.5
Areola Width	5	0.9	2.7	1.7	0.5
Areola Length	5	2.9	8.8	6.7	1.6
Female					
Carapace Length	24	16.4	43.1	31.2	8.0
Palm Length	24	4.8	24.2	11.3	5.6
Areola Width	24	1.1	2.9	1.5	0.5
Areola Length	24	3.7	11.5	2.2	2.5

#### Habitat and natural history.

**Figure 24.**
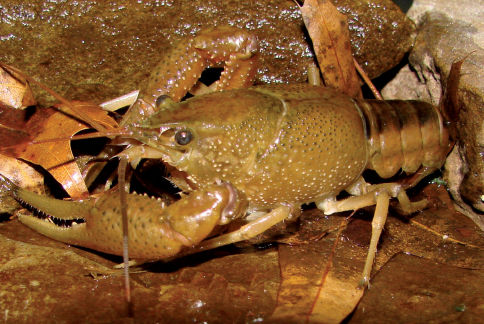
Procambarus acutus, Middle Ohio South, Mason County – WLU 04022601

Procambarus acutus (Figure 24) had not been collected from West Virginia prior to this survey. Loughman (2007) reported itslife history in the state. He found the species at only three of 18 sites surveyed in Mason County during 2004–2005. Captures in minnow traps peaked in late-March and decreased steadily until May. At this time, trapping rates were minimal and most specimens were collected during nocturnal searches by hand or with dip nets. During the summers of 2004–2005, ephemeral wetlands harboring Procambarus acutus experienced periods of drawdown leading to elevated burrowing activity. Burrow morphology for Procambarus acutus consisted of simple vertical shafts 30–40 cm deep ending in an enlarged central chamber. Chimneys present at the entrances of burrows ranged from 5 to 15 cm high.

Form I males were collected in all months between February and May, while form II males were also collected from February through April (Table 9). Females were taken in all spring months, with an increase of captures in March. Three with 4_th_ stage instars were collected on 22 March 2005. Number of pleopodal instars for each female was 72, 72 and 65, with a mean of 69. There was a positive correlation (r2 = 0.963, n = 3) between carapace length and total number of pleopodal instars. Procambarus acutus populations appear to have six distinct size cohorts although these data need to be interpreted with caution due to low sample size (Jezerinac et al. 1995).

Amplexus was observed in the field on 5 May 2005 at 22:00 h. Two amplexing pairs were observed resting on pond substrate adjacent to fallen logs. No amplexing pairs were observed away from cover objects. Several specimens also amplexed in collecting buckets within minutes of being introduced on this same day. An interspecies amplexus was observed on 5 May 2005, when a large form I male Procambarus acutus was coupled with a female Orconectes virilis for 30 minutes. In the laboratory Procambarus acutus displayed reproductive behaviors (amplexes) from early May 2005 through mid-July 2005. Crayfish associates collected with Procambarus acutus included Cambarus thomai, Cambarus bartonii cavatus, Fallicambarus fodiens and Orconectes virilis.

#### Conservation status within study area.

Based on the Marietta River Valley theory, we believe that Procambarus acutus is a native species and should be given protection. Future investigations need to focus on the Kanawha River Floodplain between Point Pleasant and St. Albans. The Moose Lodge wetland is located on the Kanawha River and is the most diverse site surveyed in this study. All major habitats along the Ohio River floodplain that have been surveyed extensively in Mason County did not yield any additional Procambarus acutus populations. When present at a site, Procambarus acutus was the dominant surface water crayfish. Conservation efforts for this species should focus on preserving habitat. The Moose Lodge wetland should be conserved for protection and monitoring, not only for Procambarus acutus populations, but also for the myriad of Marietta River relicts occurring in the wetland.

### Potential West Virginia Ohio River floodplain species

Cambarus **(Jugicambarus)** monongalensis Ortmann, 1905

Allegheny Blue Mudbug

**Figure 25.**
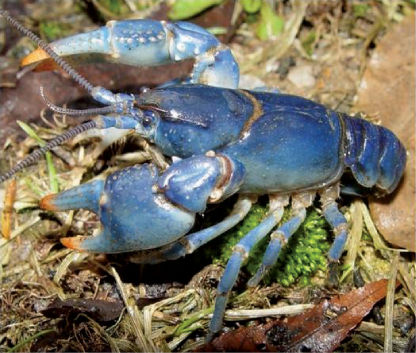
Cambarus monongalensis, Upper Ohio South basin, Ohio County, West Virginia

Cambarus monongalensis Ortmann, 1905 (Figure 25) inhabits the Upper Ohio North, Upper Ohio South, and Middle Ohio North basins (Jezerinac et al., 1995). Within these basins, Cambarus monongalensis occur in seeps, springs, roadside ditches, and creek banks in mesophytic habitats away from the floodplain. Jezerinac et al. (1995) noted that Cambarus monongalensis inhabits the Ohio River floodplain. A review of historic records indicated that specimens have been collected on hillsides adjacent to the floodplain, but not from the floodplain proper. Riparian corridors are used by this species; however, more terrestrial situations are preferred. Along the floodplain, insular Cambarus monongalensis populations likely exist, though no populations were discovered in this study. The species prefers terrestrial mesophytic situations, and microhabitats associated with floodplain systems appear to be disadvantageous for this species.

Several Cambarus monongalensis colonies have been discovered in anthropogenic habitats close to the floodplain by the primary author. Three populations in Hancock County are within 2 km of the Ohio River in exposed environments without canopy cover. Within the Upper Ohio North and Upper Ohio South basins, Cambarus monongalensis populations are present on hillsides bordering the Ohio River mainstem, but not on the floodplain proper. In the river basins in question, competition with Cambarus thomai may prevent Cambarus monongalensis from becoming established on the floodplain.

Cambarus **(Jugicambarus)** dubius Faxon, 1884

Upland Burrowing Crayfish

**Figure 26.**
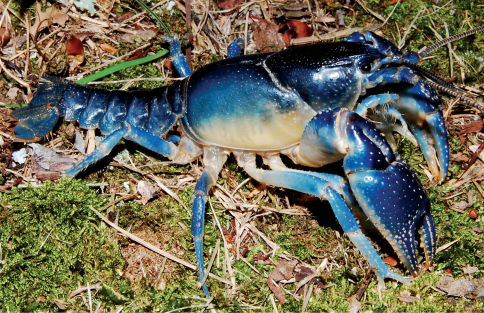
Cambarus dubius, Lower Kanawha basin, Mason County, West Virginia

Cambarus dubius Faxon, 1884 (Figure 26) populations have not been found during this study. Historic records exist for blue phase Cambarus dubius along the floodplain in Mason County (Jezerinac et al. 1995); however, Cambarus thomai was the only crayfish collected at these sites in this study. This species constructs intricate burrows in rocky soils with multitudes of ancillary tunnels that follow crevices, making collecting extremely difficult (Dewees 1972, Jezerinac et al. 1995). Although this species was not collected during this study, populations may persist along the floodplain and warrant future survey efforts.

## Discussion

### Hydrologic Watershed Faunas

Five basins compose the West Virginia Ohio River floodplain (Figure 4). From north to south they are the Upper Ohio North, Upper Ohio South, Middle Ohio North, Middle Ohio South and Lower Ohio. Basins are referred to as 8-digit watersheds as designated by government agencies. The following is a synopsis of each basin’s floodplain crayfish fauna.

#### Upper Ohio North

Four crayfish species inhabit the Upper Ohio North basin‘s floodplain (Table 14). Cambarus carinirostris is a secondary burrower that occurs throughout the basin and is present in most streams along the floodplain. It uses headwater systems, but was observed in larger streams with reduced relative abundance. Interspecific competition with Orconectes obscurus and Cambarus robustus, may limit its expansion into these environments.

**Table 14.** Major watershed distribution of West Virginia Ohio River floodplain crayfish.Single asterisk denotes presence in basin outside of floodplain. Double asterisk denotes invasive species.

Crayfish	Upper Ohio North	Upper Ohio South	Middle Ohio North	Middle Ohio South	Lower Ohio
Cambarus carinirostris	×	×	×		
Cambarus bartonii cavatus			×	×	×
Cambarus robustus	×		×	×*	×*
Cambarus thomai	×		×	×	×
Fallicambarus fodiens				×	×
Orconectes obscurus	×	×	×		
Orconectes sanbornii			×	×	×
Orconectes virilis**			×	×	
Orconectes rusticus**		×			
Procambarus acutus				×	×

Orconectes obscurus and Cambarus robustus are dominant in larger streams. Orconectes obscurus outnumbers Cambarus robustus in all streams; but Cambarus robustus specializes in colonization of slab boulders and is dominant in this microhabitat (Hamr and Berrill 1985). Orconectes obscurus and Cambarus carinirostris comprise the typical basins stream crayfish assemblage. Kings Creek, Holbert Run, and Hardin Run are stream examples with this fauna.

Cambarus thomai is the only primary burrower found in lentic habitats along the floodplain. Cambarus monongalensis can replace this species in terrestrial systems, and although it was not captured during this survey, insular populations likely inhabit the floodplain. Unlike in southern basins, Cambarus thomai is not abundant throughout the Upper Ohio North, indicating that possible limiting factors exist for this species in northern West Virginia.

#### Upper Ohio South

Four crayfish species, including an invasive species, comprise the Upper Ohio South basin fauna (Table 14). Cambarus carinirostris occupies the same niche there as in the Upper Ohio North; however, increased population densities exist in larger order streams than in Upper Ohio North populations. Compared to the Upper Ohio North, interspecific competition is limited in this basin between Cambarus carinirostris and Cambarus robustus. Cambarus carinirostris was collected more frequently in large stream channels than in marginal habitats.

Cambarus robustus and Orconectes obscurus are both tertiary burrowers occurring in the basin. Orconectes obscurus is more prevalent than Cambarus robustus, with the latter limited to the extreme southern portions of the basin in Fish Creek and its tributaries. Cambarus robustus habitat includes an abundance of slab boulders and deep pools. Orconectes obscurus is the most common tertiary burrower occurring in all major stream systems. High population densities are present in Wheeling Creek, Big Grave Creek, Little Grave Creek, and Fish Creek. Population densities of Orconectes obscurus indicate the species is successfully competing for resources with syntopic species.

No burrowing crayfishes were collected along the Upper Ohio South basin floodplain, but Cambarus monongalensis is present in terrestrial mesophytic habitats. Insular populations likely inhabit portions of the floodplain. A decrease in riparian bottomland habitat occurs within the basin. Hillsides with steep relief directly abut the Ohio River for large reaches, eliminating the potential for bottomland hardwood habitat occurrence. The lack of physical relief needed for bottomland forest may explain the lack of floodplain burrowing crayfish populations within the Upper Ohio South drainage. Historic bottomland forests present within this basin have been either altered or destroyed, and currently no contiguous tracts exist.

Orconectes rusticus, an invasive species, has populations within this basin. Populations are limited to the southern portion of the basin along the Marshall /Wetzel county lines. These populations represent potential sources for future invasions, and could spread into the Upper and Middle Ohio North basins. Monitoring is needed to see if such an invasion occurs.

#### Middle Ohio North

The Middle Ohio North basin crayfish fauna is composed of six native and one invasive species (Table 14). Northern and southern faunas merge within this basin, with several crayfish community shifts occurring. These shifts include Orconectes obscurus and Orconectes sanbornii, and Cambarus carinirostris and Cambarus bartonii cavatus, which change from a northern (Orconectes obscurus and Cambarus carinirostris) to a southern fauna (Orconectes sanbornii and Cambarus bartonii cavatus).

This is the only basin where two secondary burrowing stream forms occur but not syntopically. Cambarus carinirostris occupies Proctor Creek in extreme northern areas, while Cambarus bartonii cavatus dominates in the remaining stream systems. Both species occur in headwater streams. Cambarus carinirostris occupies larger streams within the northern regions of the basin than does Cambarus bartonii cavatus, with the latter limited to first through third order streams.

Orconectes obscurus and Orconectes sanbornii are both tertiary burrowers, which occupy the Middle Ohio North basin. Orconectes obscurus populations are shifting south and occupy stream systems where Orconectes sanbornii historically occurred. Cambarus robustus, another tertiary burrower, is also found but in reduced numbers relative to the orconectids. Within the Middle Ohio North basin, Cambarus robustus inhabits smaller streams than occupied by populations in the Upper Ohio North and Upper Ohio South basins. Orconectes virilis, an invasive species, occurs in one river embayment. This population could be a potential source for future invasions.

**Figure 27.**
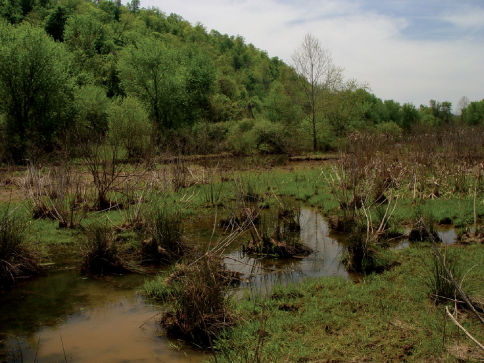
Ben’s Run, Tyler County, West Virginia at confluence with the Ohio River. Cambarus thomai and Orconectes obscurus were collected at this site.

Cambarus thomai is the sole primary burrowing crayfish species inhabiting the Middle Ohio North floodplain. Populations in New Martinsville, Wetzel County, are the most abundant and there are healthy northern populations along the floodplain. Large populations of the species are present at the Ben’s Run/Ohio River confluence (Figure 27), New Martinsville maple swamp, and ephemeral wetlands in Friendly. Relief in this part of the basin allows for well-developed bottomland habitats.

#### Middle Ohio South

Six native and one invasive species comprise the Middle Ohio South crayfish fauna (Table 14). Invasive Orconectes virilis populations reside in a critical location at the border of the Upper Ohio, Lower Kanawha, and Middle Ohio South basins. Based on their close proximity, it is highly probable that Orconectes virilis populations are present within these neighboring basins.

The crayfish fauna of this basin is similar to that of “southwestern West Virginia”. Cambarus bartonii cavatus colonizes headwater streams and Orconectes sanbornii larger streams in the basin. Cambarus thomai is the dominant primary burrower. These three species are sympatric throughout the basin, with each specializing in its own microhabitats. This assemblage is contiguous throughout the remainder of the state’s watersheds along the floodplain.

Cambarus robustus was not collected from Ohio River stream confluences, but occupies stream mainstems. The lack of confluence collections is most likely an artifact of sampling bias, since many of the reaches exhibit adequate habitat but sampling is difficult. Cambarus bartonii cavatus occurs in several lentic habitats including road rut pools and marshes fed by headwater streams. These habitats are utilized particularly by Cambarus bartonii cavatus and Cambarus thomai. For most of the watersheds these species are sympatric.

Primary and secondary burrowers occupy large sections of this basin, with the exception of Jackson County. The county’s floodplain is mostly used for agriculture, and both Cambarus thomai and Cambarus bartonii cavatus appear to respond negatively to such practices. Thus, both species within Jackson County are limited. The remaining portions of the basin have restricted amounts of agriculture, and consequently thriving Cambarus bartonii cavatus and Cambarus thomai populations.

Procambarus acutus and Fallicambarus fodiens populations occur in the southern portion of the Middle Ohio South and Lower Kanawha border. They are limited to the historic Marietta River Valley, and their absence from the central portions of the basin could be due to lack of immigration corridors. Krodel Park in Point Pleasant needs immediate conservation action; Orconectes virilis populations within Krodel Lake need to be eliminated. Given the small, isolated nature of the lake this action could be performed in an efficient manner so that future invasions via this source do not occur. In addition, laws regulating invasive species culture in West Virginia are needed to limit aquaculture production of such species.

**Figure 28.**
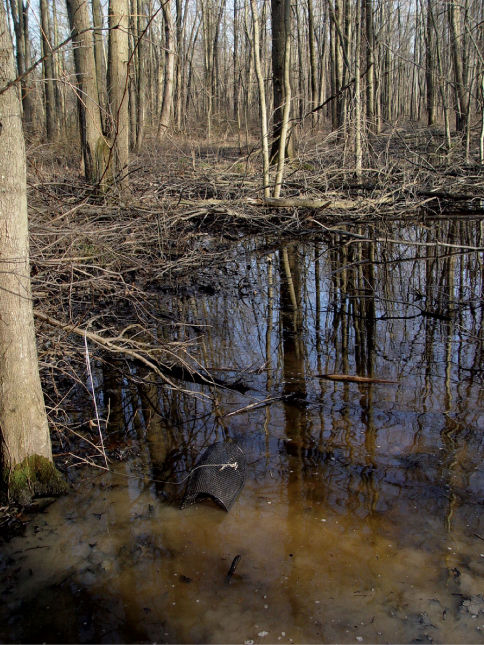
Moose Lodge Site, Mason County, West Virginia – Winter

**Figure 29.**
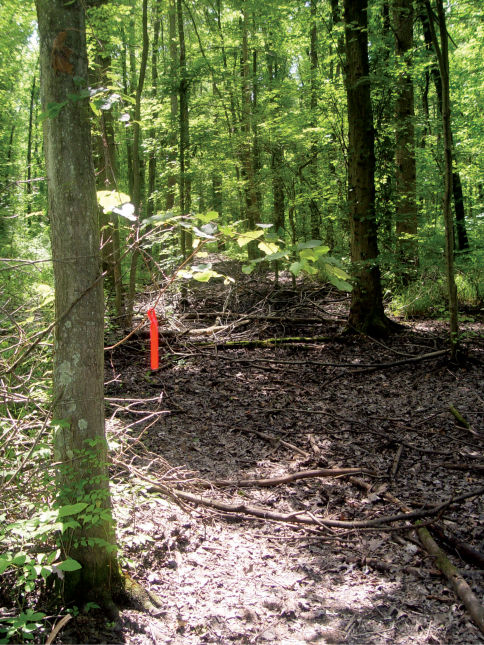
Moose Lodge Site, Mason County, West Virginia – Summer

Native crayfish populations are plentiful in ephemeral wetlands in southern portions of the basin; The Moose Lodge wetland has the most diverse native fauna along the floodplain (Figures 28 and 29). Four species, including Procambarus acutus and Fallicambarus fodiens, attain high population densities there, where Procambarus acutus populations reach their highest densities for its entire known range in West Virginia. In addition, both Cambarus bartonii cavatus and Cambarus thomai populations occupy the Moose Lodge wetland complex. This represents a rare situation, since no other known location within the state has such a diverse burrowing crayfish fauna.

#### Lower Ohio Basin

Six native crayfishes comprise the Lower Ohio basin fauna (Table 14). No invasive species are presently in the floodplain, but they do reside within the basin. Orconectes rusticus and Orconectes virilis are located sporadically throughout the basin and most likely will appear in the future along the floodplain (Jezerinac et al. 1995; Loughman et al. 2009). The need to maintain the current native composition of this fauna is of primary conservation importance.

Topographic relief along the floodplain reaches its lowest gradient within this basin, producing numerous lentic and lotic habitats conducive to floodplain crayfishes. The lotic crayfish fauna includes Orconectes sanbornii, Cambarus bartonii cavatus, and Cambarus robustus. The latteroccurred in stream mainstems, but not in tributary confluences such as those that occur in the Middle Ohio South basin. It is likely that Cambarus robustus occurs within these confluences, but sampling was insufficient. Orconectes sanbornii and Cambarus bartonii cavatus occupy all streams within the Middle South basin. Lower stream gradients persist within this basin, which benefits Orconectes sanbornii and Cambarus bartonii cavatus.Orconectes sanbornii and Cambarus bartonii cavatus occur sympatrically, with some niche partitioning among stream size. Cambarus bartonii cavatus utilizes larger ordered streams more frequently in this basin than in other northern basins.

Burrowing species reach their highest densities within this basin, particularly Cambarus thomai. Roadside ditches, maple swamps, bottomland forests, and embayments are all utilized by this species. Within lentic habitats Cambarus bartonii cavatus populations are reduced compared to Middle Ohio South populations. One potential cause for this reduction is the lack of headwater streams within the floodplain, with the majority of aquatic habitats being ephemeral lentic pools.

Procambarus acutus was collected only from northern portions of the basin. Historical range limits within the central and southern portions remain unknown and require future investigation. Fallicambarus fodiens populations are disjunct from counterparts in the Middle Ohio South basin, with one population present in the northern reaches and another in the southern portion. The northern population is the population reported by Jezerinac and Stocker (1987).

The largest Fallicambarus fodiens floodplain population occurs in Greenbottom Swamp, Greenbottom Wildlife Management Area. Greenbottom populations reach their highest densities at Hoeft Marsh. Within the marsh these populations coexist with Cambarus thomai and Cambarus bartonii cavatus. No invasive crayfish were collected from Greenbottom Wildlife Management Area, which is surprising in light of this location’s use by anglers. Concentrated sampling effort was performed within Greenbottom to locate Procambarus a acutus populations, but none were collected. Crayfish diversity in Greenbottom Wildlife Management Area is second only to that of the Moose Lodge wetland, and conservation efforts should focus on preserving this diversity.

### Conservation concerns for West Virginia Ohio River Floodplain crayfish populations

Several potential areas of imperilment exist along the floodplain, results of destruction and alteration of floodplain habitat and invasion of nonindigenous species. The following discussion is an assessment of the most relevant conservation concerns confronting Ohio River floodplain crayfishes in West Virginia. The connections between crayfish conservation concerns suggest that none of the issues is more significant than another, but each of the following requires future research.

### Habitat degradation, fragmentation, and destruction

The current thinking in conservation biology is to change emphasis of protection of individual species to protection of ecosystems (Groom et al. 2006). Crayfish conservation along the Ohio River floodplain would benefit from an integrated management plan. The most detrimental impact to floodplain crayfish populations is anthropogenic manipulation of habitat (Z. J. Loughman, personal observation). Along the floodplain, diverse crayfish faunas seem to be correlated with contiguous bottomland forest tracts or mature wetlands with buffer zones composed of heterogeneous microhabitats that reflect limited anthropogenic pressures. Positive habitat attributes are exemplified by the Moose Lodge wetland in Point Pleasant, Mason County, West Virginia, a mature bottomland forest (Figures 28 and 29).

Ephemeral wetlands provided by the Moose Lodge property comprise the most diverse crayfish assemblages present on the Ohio River floodplain. Four native species, including Cambarus bartonii cavatus, Cambarus thomai, Fallicambarus fodiens, and Procambarus acutus and the invasive Orconectes virilis, occur within the wetland. Satellite imagery analysis (Wadsworth and Treweek 1999) indicates riverine bottomland forest comprises 95% of the site with mixed stands of pin oak (Quercus palustris Müenchhausen), silver maple (Acer saccharinum L.), and black gum (Nyssa sylvatica Marsh). Bottomland forest sites, such as Moose Lodge wetland, represent an ecological dynamic unique to ephemeral wetlands. Forest canopies and the allochthonous energy contained within them are important forage for crayfishes and provide cover in ephemeral pools (Colburn 2004). These stands provide adequate nutrient cycling and shading during active hydroperiods. Forest canopies prolong hydroperiods by reducing evaporation. Hydroperiod length can be extended through late spring and early summer, enabling neonate and juvenile crayfish longer forage duration in open water prior to the construction of their initial burrows (Taylor and Anton 1998).

Periphyton growing on abscised leaves and other woody debris in pools has been shown to be a preferred forage of several crayfishes, producing larger, stronger individuals compared to animal protein or vegetative matter (Charlebois and Lamberti 1996; Chambers et al. 1990). During drawdown the duration and availability of this forage is reduced. When crayfishes leave open water environments and begin life in the burrow, there is a switch in forage type from periphyton to roots in the burrow proper and various plants and animals that wander near the entrances to burrows (Z. J. Loughman, personal observation). Forage associated with burrow existence is not as readily available as pool periphyton, which results in decreased growth and possible loss of juvenile fitness. Wetlands lacking forest canopies have shorter hydroperiods. Juvenile crayfish in these habitats enter burrows earlier than individuals occupying mature bottomland forests; thus valuable time to forage on periphyton is lost.

The importance of hydroperiod in ephemeral wetlands is reflected in the life history adaptations of burrowing crayfish. Primary burrowing crayfish generally deposit 4_th_ stage instars into surface waters in late spring to forage and mature (Hobbs 1942, 1981). As neonates and juveniles disperse they distribute themselves throughout a wetland. Mature bottomland forest pools include a variety of diverse microhabitats, providing sympatric crayfishes multiple niches to exploit. Heavily impacted ephemeral pools tend to have homogenized microhabitats, which may promote competitive exclusions among crayfish populations. The Moose Lodge bottomland wetland possesses an array of microhabitats that likely reduce competitive exclusion, allowing for the diverse fauna observed at the site.

**Figure 30.**
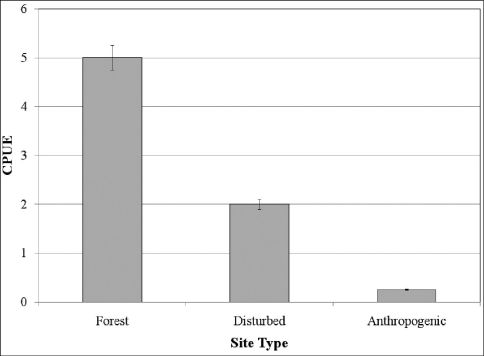
Cambarus thomai relative abundance per habitat designation.

Buffers mediate between direct contact with anthropogenic impacts (i.e., roads, industrial sites, houses) and improve site condition, providing more stable, diverse, and increased numbers of crayfish populations than those without buffers (Figure 1). These buffers range from riparian areas with herbaceous vegetation to small stands of trees with moderately impacted soil. In this study, sites with mature bottomland forests and buffer zones had the highest Cambarus thomai catch-per-unit-effort (CPUE), while sites without buffer zones that lacked canopies comprised less stable populations with reduced numbers of individuals (Figure 30). Sites without buffer zones usually had direct contact with hard surfaces that were impaired by road runoff. Increased water quantity and increased soil conductivity from hard surface runoffs (i.e., road salt and brine) have been linked to disruption during molt cycles with crustaceans (Thorp and Covich 2001). Similar issues could be impacting floodplain crayfish populations.

Agricultural impacts caused the most extreme anthropogenic stressors, including the clearing of forests and riparian buffer zones, increased usage of chemicals (i.e., pesticides and herbicides), and compacted soils. Cambarus thomai, which was the most prevalent burrowing species along the least-disturbed portions of the floodplain, were particularly impacted and reduced at sites associated with agricultural activities (Figure 30). At agricultural habitats crayfish burrowing activity ceased, with 97% of agricultural sites lacking any burrowing crayfish. Anthropogenic pressures associated with agricultural land use likely work synergistically toward the extirpation of burrowers, but specific factors may be more important than others (Z. J. Loughman, personal observation). For example, livestock foot traffic compacts regoliths and destroys burrow entrances. Grow and Merchant (1980) investigated Cambarus diogenes burrow physiochemical attributes and determined that multiple entrances to a burrow not only offer crayfish an extra exit portal but also functioned in replenishing fresh oxygen to the burrow network. As these entrance portals are destroyed by free-ranging livestock, aeration function is lost. Other direct effects include crushing of crayfish by livestock. Likewise, large amounts of nitrogenous waste are deposited within confined animal feedlots (Z. J. Loughman, personal observation)

With the conservation goal of preserving diverse floodplain crayfish populations, protection of remaining bottomland forest habitat in the Ohio River riparian corridor must occur. Less than 10 tracts of bottomland forest remain that are 100 hectares or larger in size. Sites such as Boaz Swamp, Greenbottom Swamp, McClintock Wildlife Management Area, and the Moose Lodge wetland are critical areas in need of protection. This action will preserve both crayfish diversity and multiple floral and faunal communities unique to this habitat.

### Invasive crayfish populations

Two invasive crayfishes, Orconectes rusticus and Orconectes virilis, were discovered along the Ohio River floodplain. Both species occur throughout the mid-Atlantic region and are responsible for native crayfish declines and extirpation (Loughman et al. 2009; Kilian et al. 2010; Loughman and Welsh 2010; Swecker et al. 2010). The impact these invasive populations have on primary burrowing species is largely unknown. Invasive populations carry the possibility of new pathogen and parasite introductions into a system. This situation has previously been exhibited by the decline of astacid crayfishes by cambarid introductions into European waterways (Hobbs III et al. 1989; Lodge et al. 2000a). North American primary burrowing crayfish likely have evolved alongside possible pathogens. Exposure risk to introduced Orconectes species may be minimized by habitat differences. These two groups possess decreased exposure possibilities between primary and tertiary burrowing crayfishes, which could result in juvenile primary burrowers being exposed to pathogens unique to tertiary burrowers while in river backwaters and ephemeral pools impacted by river pulses. Exposure could result in possible increase in pathnicity should a primary burrower be exposed to unique Orconectes pathogens.

Invasive orconectids impact stream dwellers more extensively than other groups (Hobbs et al. 1989; Distefano et al. 2009b; Taylor et al. 1997; Taylor et al. 2007). Species under direct threat along the Ohio River floodplain include Orconectes obscurus, Orconectes sanbornii, and Cambarus robustus. These species occupy lotic systems and share resource areas overlapping invasives. Lodge et al. (2000a, b) have shown that invasion by orconectids is two-tiered. Habitat domination causes increased native species physiological stress, as well as increased rates of predation to exposed natives (Lodge et al. 2000a, b).

Impact among Orconectes species results in the production of hybrid swarms (Butler 1988; Lodge et al. 2000a, b). Interspecies mating between native and invasive Orconectes species occurs at increased levels resulting in infertile hybrids. Butler (1988) observed this interaction between Orconectes rusticus and Orconectes sanbornii in Ohio and it is likely this interaction occurs within West Virginia populations.

Orconectes rusticus populations were collected in Ohio River backwaters in Marshall county. All Orconectes rusticus sites were heavily impacted by land use (i.e., nutrient rich agriculture and channelization) and had direct connections to the Ohio River; however, stream confluences within 1 km north and south of these sites did not harbor Orconectes rusticus populations. Orconectes rusticus reached high densities in these backwater habitats, feeding on the abundance of macrophytes and periphyton. Backwater microhabitats utilized by Orconectes rusticus include shallow, littoral regions. This microhabitat provides abundant forage and refugia along large river backwaters. The invasion dispersal occurs through smaller stream confluences with the Ohio River. Understanding the dispersal relationship between invasive crayfish and large river system corridors and smaller stream confluence connections is an important research need.

### Natural and Life History Study Needs

Successful conservation of imperiled species is directly correlated with a fundamental understanding of life history parameters and species ecological needs (Groom et al. 2006). Crayfish life history diversity is poorly understood. Elucidating different ecological patterns utilized by crayfishes as life history parameters is an action needed to better understand the biology of these animals (Taylor et al. 1996; Schuster 1997; Taylor et al. 2007). Based on a pool of research, crayfish do not have a single life history strategy (Simon et al. 2005; Loughman et al. 2009). This is evident in the diversity of speciation events that have occurred within the cambaridae in North America (Hobbs 1942; Hobbs et al. 1969; Hobbs 1981). Many of these events are proving to be cryptic in nature, and only now are being described via genetic analysis (Taylor et al. 2007). One causal agent of speciation is niche occupation and specialization; an act that has occurred repeatedly with crayfish in eastern North America (Hobbs 1969). Speciation is directly associated with a change in ecological needs in response to environmental stressors acting on gene flow such as geographic barriers, which leads to diversity of natural histories among closely related taxa (Groom et al. 2006).

Organisms respond differentially to environmental stressors, and in the case of genetic lineages, different approaches to ecological adaptation can result in the creation of unique forms. Life history models (i.e., primary, secondary, and tertiary burrowers as proposed by Hobbs (1981) have proven reliable and enable niche groupings among species utilizing similar life histories. However, many crayfishes remain understudied and in many instances unknown. An understanding of different life histories can only increase our knowledge of ecological needs of crayfishes and allow more efficient conservation management.

Future Ohio River floodplain conservation efforts should focus on elucidating specific aspects of crayfish natural histories. Forage preference, macro- and micro-habitat usage, inter- and intra-specific interactions, behavioral ecology, and fecundity are just a few natural history parameters in need of determination for many crayfish species. Most Ohio River floodplain crayfish basic life history information such as life span, total possible fecundity, recruitment rates, and foraging preference is unknown. The only species within the floodplain that have received considerable ecological research include Orconectes rusticus and Orconectes virilis (Capelli 1982; Chambers et al. 1990). The focus of these efforts was to better understand their destructive nature.

Most species, especially non-invasive taxa, have received minimal consideration. One reason for these differences in attention is that one or two landmark studies have been performed on species whose life histories are then broadly applied to crayfish behavioral groups (i.e., primary or secondary burrowers). For instance, Grow and Merchant (1980) investigated the abiotic realm of Cambarus diogenes, a primary burrower, on the coastal plain of Maryland. The ecological theater for Cambarus diogenes on the coastal plain is quite different than that of Cambarus thomai on the Ohio River floodplain. Automatically assuming that the needs of these closely related species are the same may mislead an investigator. Studies on a variety of primary burrowing species should be conducted prior to generalizing the ecological requirements of this behavioral group.

Details of Ohio River floodplain crayfish natural history may provide linkages to the specific mechanisms causing species decline. Once specific mechanisms are identified, conservation actions can be formulated. Information can be shared among astacologists and government agencies responsible for protecting non-game wildlife. These data can assist in the creation of management plans and their implementation; thus ensuring a rich floodplain crayfish fauna for future generations.

## Supplementary Material

XML Treatment for Cambarus (Cambarus) carinirostris

XML Treatment for Cambarus (Cambarus) bartonii cavatus

XML Treatment for Cambarus (Puncticambarus) robustus

XML Treatment for Cambarus (Tubericambarus) thomai

XML Treatment for Fallicambarus (Creaserinus) fodiens

XML Treatment for Orconectes (Crockerinus) obscurus

XML Treatment for Orconectes (Crockerinus) sanbornii

XML Treatment for Orconectes (Gremicambarus) virilis

XML Treatment for Orconectes (Procericambarus) rusticus

XML Treatment for Procambarus (Ortmannicus) acutus
